# *Trichoderma afroharzianum* TRI07 metabolites inhibit *Alternaria alternata* growth and induce tomato defense-related enzymes

**DOI:** 10.1038/s41598-024-52301-2

**Published:** 2024-01-22

**Authors:** Bassant Philip, Said I. Behiry, Mohamed Z. M. Salem, Mostafa A. Amer, Ibrahim A. El-Samra, Ahmed Abdelkhalek, Ahmed Heflish

**Affiliations:** 1 Agricultural Botany Department, Faculty of Agriculture (Saba Basha), Alexandria University, Alexandria, 21531 Egypt; 2 Forestry and Wood Technology Department, Faculty of Agriculture (El-Shatby), Alexandria University, Alexandria, 21545 Egypt; 3 Plant Protection and Biomolecular Diagnosis Department, ALCRI, City of Scientific Research and Technological Applications, New Borg El Arab City, 21934 Egypt

**Keywords:** Biological techniques, Chemical biology, Microbiology

## Abstract

Identifying a viable substitute for the limited array of current antifungal agents stands as a crucial objective in modern agriculture. Consequently, extensive worldwide research has been undertaken to unveil eco-friendly and effective agents capable of controlling pathogens resistant to the presently employed fungicides. This study explores the efficacy of *Trichoderma* isolates in combating tomato leaf spot disease, primarily caused by *Alternaria alternata*. The identified pathogen, *A. alternata* Alt3, was isolated and confirmed through the ITS region (OQ888806). Six *Trichoderma* isolates were assessed for their ability to inhibit Alt3 hyphal growth using dual culture, ethyl acetate extract, and volatile organic compounds (VOCs) techniques. The most promising biocontrol isolate was identified as *T. afroharzianum* isolate TRI07 based on three markers: ITS region (OQ820171), translation elongation factor alpha 1 gene (OR125580), and RNA polymerase II subunit gene (OR125581). The ethyl acetate extract of TRI07 isolate was subjected to GC–MS analysis, revealing spathulenol, triacetin, and aspartame as the main compounds, with percentages of 28.90, 14.03, and 12.97%, respectively. Analysis of TRI07-VOCs by solid-phase microextraction technique indicated that the most abundant compounds included ethanol, hydroperoxide, 1-methylhexyl, and 1-octen-3-one. When TRI07 interacted with Alt3, 34 compounds were identified, with major components including 1-octen-3-one, ethanol, and hexanedioic acid, bis(2-ethylhexyl) ester. In greenhouse experiment, the treatment of TRI07 48 h before inoculation with *A. alternata* (A3 treatment) resulted in a reduction in disease severity (16.66%) and incidence (44.44%). Furthermore, A3 treatment led to improved tomato growth performance parameters and increased chlorophyll content. After 21 days post-inoculation, A3 treatment was associated with increased production of antioxidant enzymes (CAT, POD, SOD, and PPO), while infected tomato plants exhibited elevated levels of oxidative stress markers MDA and H_2_O_2_. HPLC analysis of tomato leaf extracts from A3 treatment revealed higher levels of phenolic acids such as gallic, chlorogenic, caffeic, syringic, and coumaric acids, as well as flavonoid compounds including catechin, rutin, and vanillin. The novelty lies in bridging the gap between strain-specific attributes and practical application, enhancing the understanding of TRI07’s potential for integrated pest management. This study concludes that TRI07 isolate presents potential natural compounds with biological activity, effectively controlling tomato leaf spot disease and promoting tomato plant growth. The findings have practical implications for agriculture, suggesting a sustainable biocontrol strategy that can enhance crop resilience and contribute to integrated pest management practices.

## Introduction

*Alternaria alternata* (Fr.) Keissler, a widely distributed soil-borne fungus, poses a significant threat to numerous economically vital plants, including solanaceous crops like potato, tomato, and tobacco^1^. Its impact extends beyond traditional hosts, causing leaf spot in diverse plants such as cast iron plants^2^, Rose of Sharon^3^, and Okra^4^. With a global presence, this disease has persistently afflicted Egyptian tomato crops over the years^5^. The prevalence of warm and humid conditions further exacerbates its impact, potentially resulting in *Alternaria* black spots grow larger, limiting the photosynthetic area, producing defoliation, and accelerating leaf loss^6–8^. Controlling this fungal disease caused by the *Alternaria* pathogen proves challenging due to its broad host range. Considering the acknowledged economic significance, the management of *A. alternata* primarily involves the application of fungicides to mitigate losses^9^. The utilization of fungicides (such as mancozeb, copper oxychloride, carbendazim + mancozeb, carbendazim, thiophanate methyl) constitutes a component of the chemical management approach for leaf spot disease^10^. Nonetheless, the efficacy of fungicides in treating fungal infections is constrained due to the emergence of more resistant strains of pathogens^11,12^. Furthermore, the use of fungicides can pose risks to both human well-being and the natural ecosystem^13^.

Biological control is seen as a viable, environmentally sustainable option due to the adverse ecological impacts associated with the employment of fungicides. Biological control agents like *Trichoderma* species have been found to effectively impede the progression of infections by employing various specialized mechanisms such as parasitism, antibiosis, and competition for resources and spatial occupancy within the rhizosphere zone^14–19^. *Trichoderma* species have been utilized with the goal of alleviating the proliferation of phytopathogenic fungi and the promotion of plant development in diverse field and vegetable crops^20,21^ such as cucumber^22^, wheat^23^, common bean^24,25^, potato^26^, tomato^27,28^ and groundnut^29,30^. The efficiency of *Trichoderma* is ascribed to its quick development and capacity to withstand unfavorable environmental conditions^31^. The antagonistic and mycoparasitic actions of *Trichoderma* on plant pathogens, namely through hyperparasitism, are regarded as crucial in diminishing the severity of diseases^32,33^. *Trichoderma* produces cell-wall-degrading enzymes (CWDEs) such chitinases, glucanases, and proteinases during the hyperparasitic phase. These enzymes damage the cell wall of the plant pathogen^34,35^.

The efficacy of *Trichoderma* strains in biocontrol has been extensively demonstrated, as evidenced by their ability to produce a diverse range of metabolites such as enzymatically active proteins, small molecules associated with fungal or plant cell walls, and other secondary metabolites, stimulate the plant’s defensive systems against infections^30,36^. *Trichoderma* species have the capacity to synthesize several volatile organic compounds (VOCs), such as pyrones and sesquiterpenes, 3-octanone, 1-octen-3-ol, 6-pentyl-a-pyrone, 3-octanol, and 1-octen-3-one, which exhibit toxic effects against fungi and bacteria^37,38^. A multitude of microbial species emit VOCs that impede the progression of infections or alter them in an atypical manner, hence diminishing their ability to engage with plant hosts^39,40^. Further, researchers have observed a large increase in intracellular metabolites such as l-proline, maleic acid, d-fructose, mannitol, and butane in potent *Trichoderma* fusants. These metabolites are believed to have biocontrol and stress-tolerant properties^41,42^.

Previous studies have employed polymerase chain reaction (PCR) to identify *Alternaria* spp. in tomatoes using ribosomal internal transcribed spacer (ITS) DNA sequence analysis^43,44^. According to Pavón et al.^43^, the ITS region sequencing revealed that *Alternaria solani* and *A. alternata* were identified as the two most detrimental infections recovered from tomato. The ITS, RNA polymerase II (*rpb*2) and translation elongation factor 1 (*tef*1), genes are highly effective in identifying species within the *Trichoderma* genera^45^. The molecular identification of *Trichoderma hamatum* strain Th23, which was obtained from the roots of tomato plants, was conducted using phylogenetic analysis utilizing the ITS, *tef*1, and *rpb*2 gene sequences^46^.

Hence, the objective of the present investigation was to evaluate the inhibitory effect of *Trichoderma* sp. on the occurrence of leaf spots on tomato plants induced by *A. alternata* under both laboratory (in vitro) and greenhouse (in vivo) conditions. The effects of *Trichoderma afroharzianum* on various growth indices, oxidative and antioxidant enzymes, chlorophyll content, phenolic content, and total protein content were investigated. Additionally, the study of *T. afroharzianum* and its volatile organic compounds (VOCs) using gas chromatography-mass spectrometry (GC–MS) and high-performance liquid chromatography (HPLC) of tomato leaves was also conducted.

## Materials and methods

### Isolation of leaf spot pathogen from tomato plants

The samples exhibiting symptoms such as leaf spots on tomato plants were gathered from Rashid City, located in the El-Behera governorate of Egypt. The geographical coordinates of the collection site were recorded as 31.403354 latitude and 30.397880 longitude. In the isolation protocol, the tomato leaves were fragmented into small segments measuring approximately 5 mm by 5 mm. These fragments were subjected to surface sterilization using a 2% sodium hypochlorite solution for 2 min. Subsequently, they were treated with 70% ethanol for 30 s. Finally, the fragments were thoroughly washed several times (specifically, two to three times) using sterile dH_2_O. The minuscule pieces were carefully positioned onto Petri dishes with potato dextrose agar (PDA) media and thereafter subjected to incubation at 25 °C ± 2 for 5 days. The fungal culture was successfully isolated and afterward transferred onto PDA slants, which were stored at 4 °C for subsequent investigations^47,48^.

### Isolation of *Trichoderma* from tomato rhizosphere

Soil samples from the rhizosphere of tomato plants in healthy growth zones were obtained. The cultivation of *Trichoderma* and the utilization of selective medium, namely TME^49^ and TSM^50^, were employed in this study. The fungal cultures obtained by the single spore isolation method^51^ were purified and thereafter maintained on PDA slants for additional experimental procedures.

### Morphological and molecular identification of isolated *Alternaria *and* Trichoderma*

The identification of isolated fungi was conducted based on their morphological and molecular features. The molecular typing of the pathogen was conducted using the internal transcribed spacer (ITS) region. The primer sequences employed for the ITS region were ITS1 and ITS4, and the PCR reactions were conducted using the methodology described in a previous study^52^. To identify the bioagent, Carbone et al.^53^ and Abdelkhalek et al.^54^, employed three molecular markers for *Trichoderma* isolate: the ITS region, the translation elongation factor alpha 1 (*tef*1) gene, and the RNA polymerase II subunit (*rpb*2) gene. In the PCR reactions, 1 µL of each primer pair (10 pmol), 20 µL of TOPsimple™ PCR PreMIX-nTaq (Enzynomics Inc., Yuseong-gu, Daejeon, Republic of Korea), 3 µL of fungus DNA, and 26 µL of molecular-grade water were combined. Cycling conditions were implemented using a thermal cycler TC-PRO (Boeco, Germany): an initial step at 94 °C for 4 min, followed by 35 cycles at 95 °C for 1 min (55 °C for ITS; 45 s, 62 °C for *rpb*2; 45 s, 57 °C for *tef*1; 60 s), and 72 °C each for 1 min, with a final extension step at 72 °C^55^. The PCR amplification samples underwent sequencing at Macrogen Inc. (Seoul, Korea), and the NCBI-BLAST tool was employed to conduct a comparative analysis between the obtained sequences and those present in the GenBank database, therefore verifying their identification. The alignment of nucleotide sequences was performed utilizing the MEGA 11 program^56^, which was employed for editing and curating the sequences to generate phylogenetic trees. The potential phylogenetic relationships among *A. alternata* isolates and *Trichoderma* species were determined using the maximum-likelihood (MLL) technique for ITS, *tef*1 and *rpb*2 genetic markers sequence data, employing MEGA 11. Gap/missing data treatment involved adjusting the entire deletion, and the initial MLL tree was automatically modified. The MLL trees obtained underwent evaluation through bootstrap analysis with 1000 replications^56,57^.

### Effect of *Trichoderma* isolates against the radial growth of *Alternaria* pathogen in vitro

A total of six isolates belonging to *Trichoderma* spp. were examined in this section. These isolates were obtained from the rhizosphere soil of tomato plants. The objective was to evaluate their efficacy against the pathogen responsible for tomato leaf spot. Various methods were employed to assess the efficiency of these isolates.

### Dual culture technique

The efficacy of six *Trichoderma* isolates in controlling the pathogen associated with tomato leaf spot was evaluated using a dual-culture methodology^58,59^. The isolated pathogen and *Trichoderma* culture mycelial with a diameter of 5 mm were positioned in the Petri plates across from one another at an equal distance from the edge. On one side of the PDA-containing petri dish, antagonists were placed. A disc from the pathogen culture was positioned on the other side of the Petri plates and incubated at 25 °C ± 2^60^. Petri dishes inoculated with fungal pathogen were only used as control. The experiment trail was repeated 3 times. The inhibition percentage of pathogen growth was calculated according to following formula: $\mathrm{Inhibition }\,(\mathrm{\%})= [({\text{C}}-{\text{T}})/{\text{C}}]\times 100$, where C represents the control and T represents the development of the pathogen in the treated plates with antagonistic isolates^61^.

### Ethyl acetate extract of *Trichoderma* isolates and its antifungal activity

*Trichoderma* isolates were grown on 100 mL of potato dextrose broth (PDB). The inoculated cultures were incubated at 25 °C ± 2 for 2 weeks under stirring (100 rpm) in INCU-SHAKER shaking incubator (Benchmark Scientific, Inc., Sayreville NJ, USA).

After 2 weeks, the fungal cultures were centrifuged at 6000 rpm for 10 min, then to ensure the filtration process, spores and mycelia of *Trichoderma* isolates were removed from PDB culture by filtration with Whatman no.1 filter paper. The rested broth media (culture filtrate) were used to extract the antagonistic metabolites by mixing the culture filtrate with ethyl acetate (1:1 v/v). Two clear immiscible layers were developed after the mixture had been thoroughly stirred for 10 min and allowed to sit for 5 min^62^. Using a separating funnel, the top layer of the solvent, which included the extracted biomolecules, was separated. The ethyl acetate extract was concentrated to small volume by evaporating the solvent in a rotary evaporator under vacuum^63^. After the ethyl acetate evaporated, brown gum was presented. After that, the crude extract was kept at 4 °C.

The antifungal activity of the prepared extract against the pathogen was evaluated using the poisoned food technique. After the extract was reconstituted (4 mg/mL) in dimethyl sulfoxide (DMSO) in 20 mL PDA, a 5 mm disc of the pathogen was put in the center of each Petri plate. Once the control plate reached the margins, the radial growth of pathogen was measured. The inhibitory effect were calculated compared to the control^64^.

### Volatile compounds emitted from *Trichoderma*

The sealed plate technique^65^ was used to examine the effectiveness of volatiles generated from *Trichoderma* isolates in inhibiting the growth of leaf spot pathogen. A mycelial plug (0.5 cm in diameter) was plugged from a 5-day-old *Trichoderma* culture plate and put in the center of PDA plate. The pathogen PDA plate was inoculated 48h before proceeding to the next step. The pathogen Petri dish led, and the antagonistic isolate was removed, and the two bottom plates were then joined by Parafilm. In the control, only the PDA medium was placed in the bottom plate (without the antagonistic isolate). Three replicates of the experiment were run. The tested plates were incubated for 5 days at 25 ± 2 °C. Plant pathogen mycelial diameters were measured in the control and the inverted plate and converted to an inhibitory percentage %^65^.

The inhibition percentage of the pathogen was calculated for all the tested methods using the formula: $\mathrm{Inhibition }\,(\mathrm{\%}) = [(\mathrm{rc }-\mathrm{ rt})/{\text{rc}}] \times 100$, where rc is the radial growth of the plant pathogen on the control plate, while rt is the radial growth of the plant pathogen on the treated plate.

### Gas chromatography–mass spectroscopy fractionation (GC–MS) analysis

The bioactive constituents within the cell-free supernatant of *Trichoderma* culture filtrate were elucidated and characterized through gas chromatography mass spectroscopy (GC–MS) analysis. For this purpose, the ethyl acetate extract was obtained as previously stated in “Effect of *Trichoderma* isolates against the radial growth of *Alternaria* pathogen in vitro” section. The resultant residues were subjected to GC–MS analysis using an Agilent 7000D instrument (Santa Clara, CA, USA) and the program conditions were programed according to Khamis et al.^66^.

Furthermore, the volatile organic compounds (VOCs) present in the *Trichoderma* sample were extracted using solid phase microextraction (SPME) at 40 °C for 20 min. The extracted VOCs were then injected into a GC–MS system (Agilent Technologies) equipped with a gas chromatograph model 7890B and a mass spectrometer detector model 5977A (Agilent technologies, Santa Clara, CA, USA)^67^.

### Effect of *Trichoderma* against *Alternaria* leaf spot under greenhouse conditions

Based on a pot experiment conducted in a controlled greenhouse environment (temperature: 28 °C; relative humidity: 75%; photoperiod: 14 light/10 dark h), the impact of the best antagonistic isolate on the activity of the *Alternaria* pathogen and plant growth was assessed. Four-week-old tomato seedlings were transplanted into plastic pots (20 cm in diameter) with 1 kg of sterile soil that was evenly divided between clay, sand, and peat moss. 10 mL of bioagent inoculum at a concentration of 1 × 10^7^ spores/mL was sprayed into each pot after the seedlings had been transplanted for 5 days^68^. To avoid the drop of *Trichoderma* droplets onto the surface of the pot soil, the treated pot soil was covered when the plants were sprayed. The treatments were carried out in the greenhouse with 3 replicates and a 10 mL inoculum of the pathogen (1 × 10^5^ spores/mL), as shown in Table 1. The fungicide used in this study was Ridomil Gold MZ 68%WG (4% Mefanoxam + 64% Mancozeb as active substance) at a recommended concentration of 2 g/L water.

**Table 1:** The treatments used in the greenhouse experiment.

Parameter code	Treatments
A1	Tomato plants sprayed with sterilized dH_2_O (negative control)
A2	Tomato plants inoculated with pathogen only (positive control)
A3	Tomato plants sprayed with bioagent before 48 h of inoculation with pathogen
A4	Tomato plants sprayed with bioagent after 48 h of inoculation with pathogen
A5	Tomato plants sprayed with Ridomil, fungicide before 48 h inoculation with pathogen
A6	Tomato plants sprayed with Ridomil, fungicide after 48 h of inoculation with pathogen

The plants are screened for disease severity (DS) 1 month after transplantation using a 0–5 scale based on the percentage of leaf area covered by necrotic lesions^69^. After observing tomato plants, the disease incidence (DI) was determined using the formula below:

$$\mathrm{DI \% }= \left[\frac{\mathrm{\Sigma \,of \,observed \,numerical \,rating}}{\left({\text{Max}}.\,\mathrm{ disease\, rating }\times \mathrm{ Total\, number \,of \,observed\,plants}\right)}\right]\times 100.$$

The plants were also employed to evaluate the impact of the bioagent on various growth parameters, including shoot height, root length, shoot and root fresh weight (g), shoot and root dry weight (g), and total chlorophyll content, which was quantified using SPAD-502 Plus (Tokyo, Japan)^27^.

### Determination of enzymes activity in tomato leaves

To prepare the tomato plant extract for enzyme assays, frozen leaves (1 g fresh mass) were ground using liquid nitrogen. Subsequently, a chilled extraction buffer consisting of 3 mL of 50 mM potassium phosphate (prepared by combining monopotassium phosphate and dipotassium phosphate, pH 7.5) was employed for material extraction. The crude extract was derived from the supernatants obtained after centrifugation of the extract at 12,000 rpm and 4 °C for 30 min. The supernatant was preserved at − 80 °C in a tube for subsequent measurement of various antioxidant enzyme activities^70^. All absorbance values were measured using a UV-1900 BMS (Waltham, MA, USA) spectrophotometer. Activity assays for peroxidase^71,72^, superoxide dismutase^73^, catalase^74^, polyphenol oxidase^75^, lipid peroxidation^76^, hydrogen peroxide^77^, total protein^70^,and total phenolic content^78^ in tomato plant leaves were conducted as per the methods detailed in the Supplementary Material.

### HPLC analysis of tomato collected leaves extract

HPLC inspection was performed using an Agilent 1260 series. A column called Eclipse C18 (4.6 mm × 250 mm i.d., 5 µm) was used for the separation. The mobile phase comprises water (A) and 0.05% trifluoroacetic acid (CF_3_COOH) in acetonitrile (B), was delivered at a constant flow rate of 0.9 mL/min. Standard compounds were used for HPLC system calibration, followed by the injection of samples (5 μL) into the system at a column temperature of 40 °C. To achieve effective separation, a gradient elution program was implemented, involving distinct phases over 20 min. The separation conditions can be found in previous work^66^. The separated compounds were detected by a UV–Visible detector at 280 nm, generating a signal proportional to their concentrations. The resulting chromatogram displayed the retention time and peak area for identification and quantification. HPLC analysis confirmed the presence of target compounds in the samples at expected concentrations, with validated precision and accuracy, underscoring the method’s suitability for characterizing complex mixtures and determining compound purity.

### Statistical analysis

All experiments were statistically analyzed by one-way ANOVA except the data on the effect of ethyl acetate extract of different *Trichoderma* isolates (Tri 1–Tri 6) against pathogen growth, which was done by two-way ANOVA using the CoStat software version 6.303 (CoHort Software, Monterrey, CA, USA). Comparisons among means were determined with the least significant difference (LSD) test at a *p* ≤ 0.05 level of probability.

### Ethics approval and consent to participate

This study is complied with relevant institutional, national, and international guidelines and legislation. This study does not contain any studies with human participants or animals performed by any of the authors.

## Results

### Isolation and identification of the leaf spot pathogen

**Figure 1:**
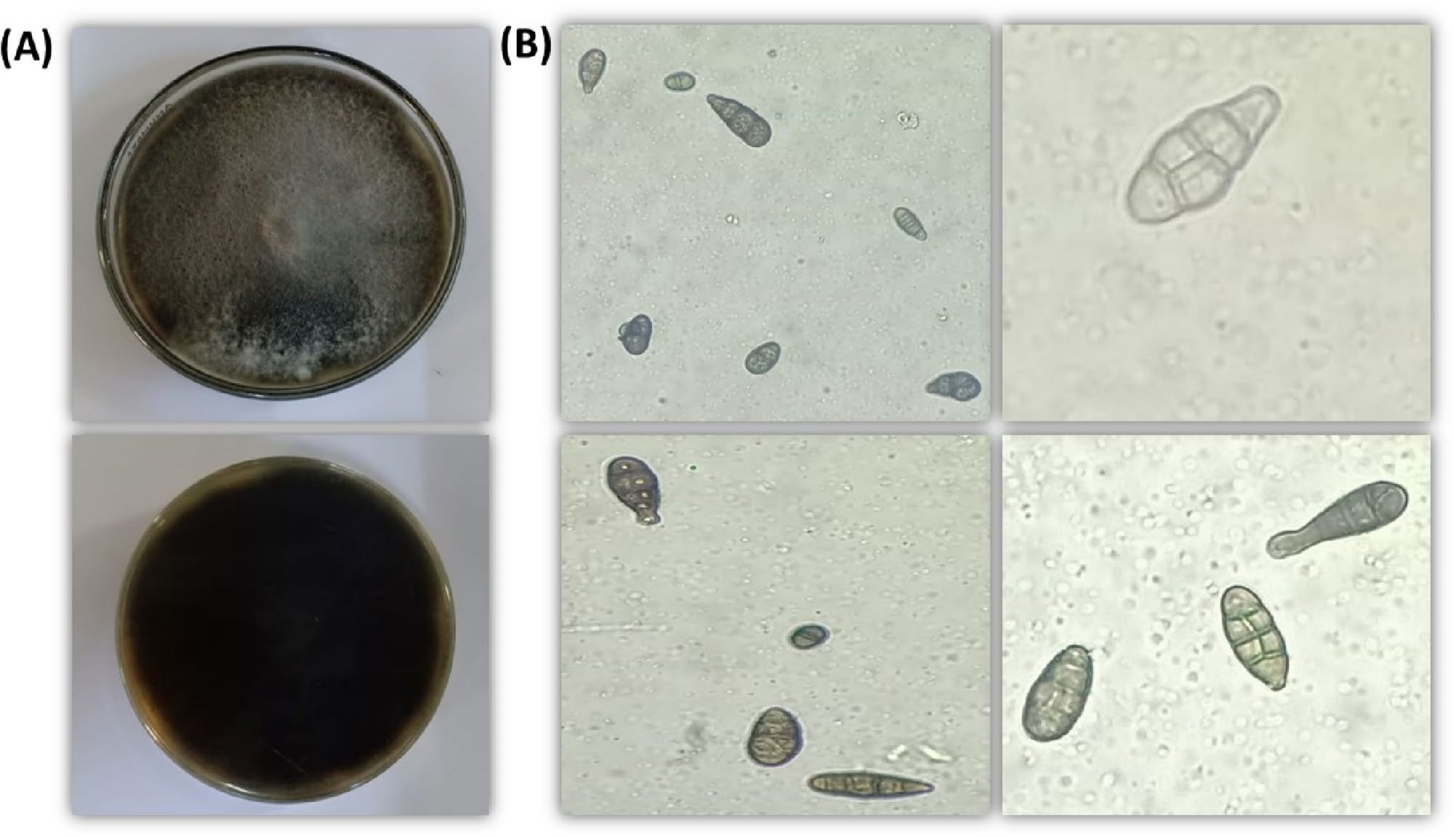
(**A**) *Alternaria alternata* growth in a Petri dish and (**B**) septate conidium-bearing conidiophore were photographed under a 40× light microscope. Scale bar = 50 μm.

The fungal strain that caused tomato leaf spot symptom was found to be the same in plants that had been infected in the field. The fungal isolate was subjected to morphological and molecular analyses to determine its identity. In order to ascertain the identity of the pathogen at the morphological level, the fungus was cultivated on a plate containing PDA medium. Subsequently, all observable characteristics were meticulously documented, including the smooth texture and the coloration, which was described as either deep or olivaceous. The conidia seen in this study were found to be solitary, exhibiting a color range from brown to olivaceous brown. These conidia were characterized by their straight or ellipsoidal tapering shape. Additionally, both transverse and longitudinal septation were observed in the conidia, as depicted in Fig. 1, panels A and B. The phenotypic traits exhibited by the pathogen provide confirmation of its classification at the genus level as *Alternaria* sp.

In order to validate the morphological identification, the molecular technique known as the inter-transcript spacer region (ITS) was employed. The ITS sequence obtained was subjected to a molecular identification process by blasting it through the GenBank portal. This analysis led to the identification of the isolated pathogen as *Alternaria alternata* Alt3, which was assigned the accession number OQ888806.

**Figure 2:**
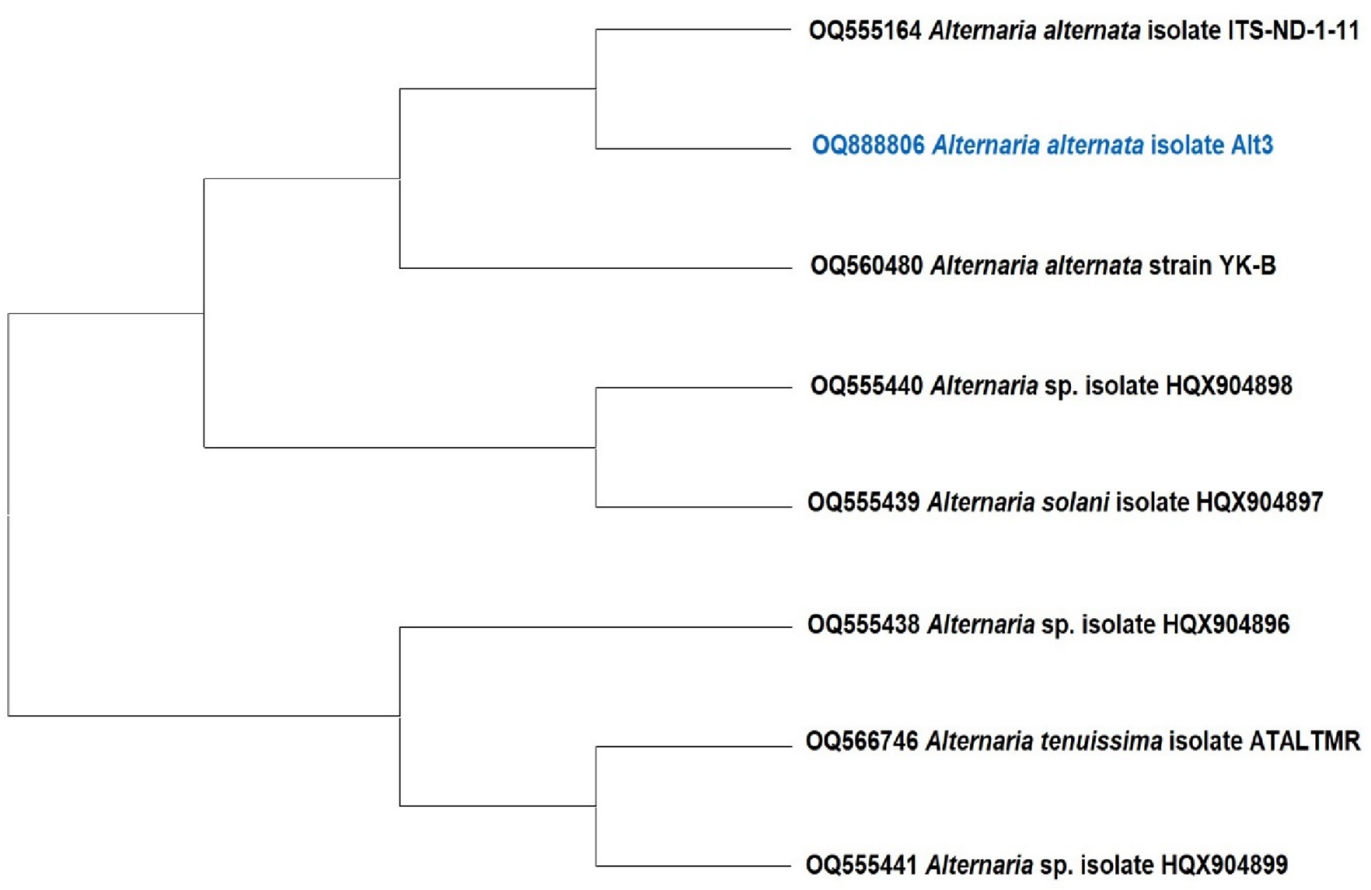
A phylogenetic tree was generated using the eight most parsimonious ITS sequences in comparison to the isolated *Alternaria alternata* Alt3 (OQ888806) sequence.

The ITS phylogenetic tree analysis revealed a complete nucleotide sequence similarity of 100% between our isolate Alt3 and *A. alternata* isolates with accession numbers OQ555164 and OQ560480, originating from China. The minimal level of nucleotide sequence similarity observed between *A. solani* fungus from China (OQ555439) and *Alternaria* sp. isolate from China (OQ555440) is depicted in Fig. 2.

### Isolation and identification of *Trichoderma* isolate

Based on established taxonomic phenotypic criteria, the examination of the isolated *Trichoderma* isolates obtained from the roots of tomato plants exhibited morphological characteristics consistent with the attributes associated with the *Trichoderma* genus. The organism generated unicellular, spherical conidia that exhibited either a smooth or rough surface texture and possessed a green coloration illustrated in the Supplementary Results (Fig. S1). Solitary phialides or aggregated phialides emerged from small terminal clusters at a perpendicular angle of 90 degrees from the conidiophore on elongated, branching, and incomplete conidiophores.

**Figure 3:**
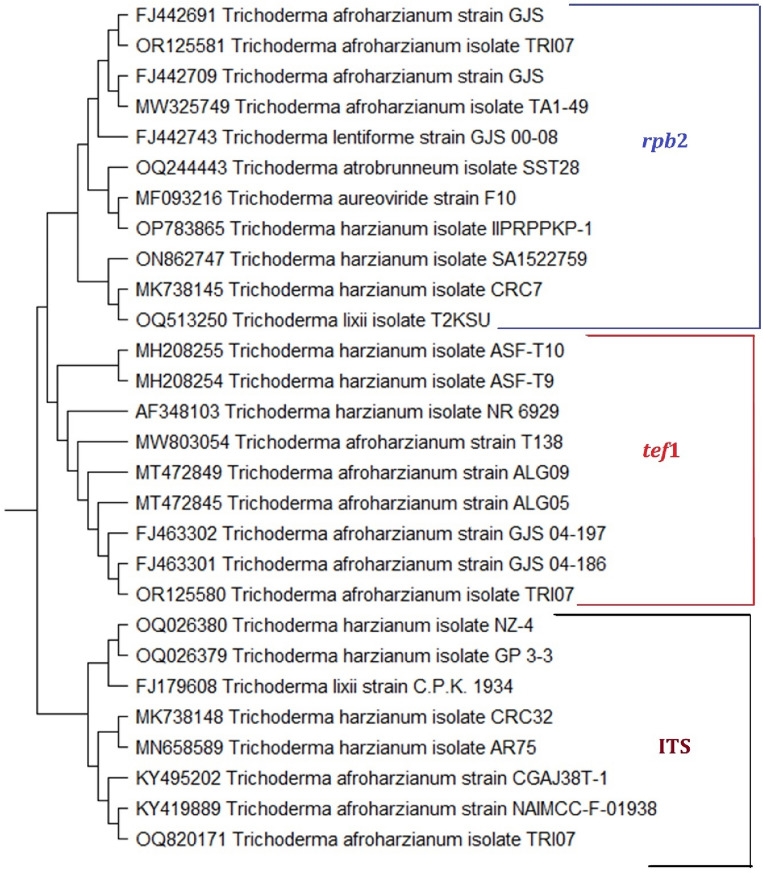
A phylogenetic tree indicates the relationship between *Trichoderma afroharzianum* isolate TRI07 and closely related *Trichoderma* isolates from GenBank, based on a partial sequence of the DNA markers ITS, *tef*1 and *rpb*2. The tree was generated by MEGA 11 software.

By amplifying PCR amplicons of the ITS primer of about 600 bp, the *tef*1 gene of about 400–600 bp, and the *rpb*2 gene amplified as a band of about 1200 bp, the *Trichoderma* isolate’s morphological identity was verified using PCR techniques. The three amplified marker partial sequences ITS, *tef*1, and *rpb*2 were acquired and uploaded to NCBI GenBank and assigned to *Trichoderma afroharzianum* isolate TRI07 with accession numbers OQ820171, OR125580, and OR125581, respectively. In a comparison of the generated ITS nucleotide sequences of the TRI07 isolate using isolates from GenBank, it was shown that there was the greatest genetic homogeneity with 100% of the ITS sequence with *T. afroharzianum* with accession numbers (KY495202 and KY419889) from Nigeria and India, respectively (Fig. 3). Comparing the *tef*1 gene of the *T. afroharzianum* TRI07 isolate with the GenBank-identified isolates demonstrated that the highest genetic homogeneity was 100% of the *tef*1 sequence with *T. afroharzianum* with accession numbers FJ463301 and FJ463302 from Peru. Also, comparing the *rpb*2 gene of TRI07 isolate using isolates from GenBank-identified databases, it was shown that the highest genetic homogeneity was 100% of the *rpb*2 sequence with *T. afroharzianum* with accession numbers FJ442691 from Peru (Fig. 3).

### Effect of *Trichoderma* isolates on the growth of *A. alternata* in vitro

#### Dual culture method

**Figure 4:**
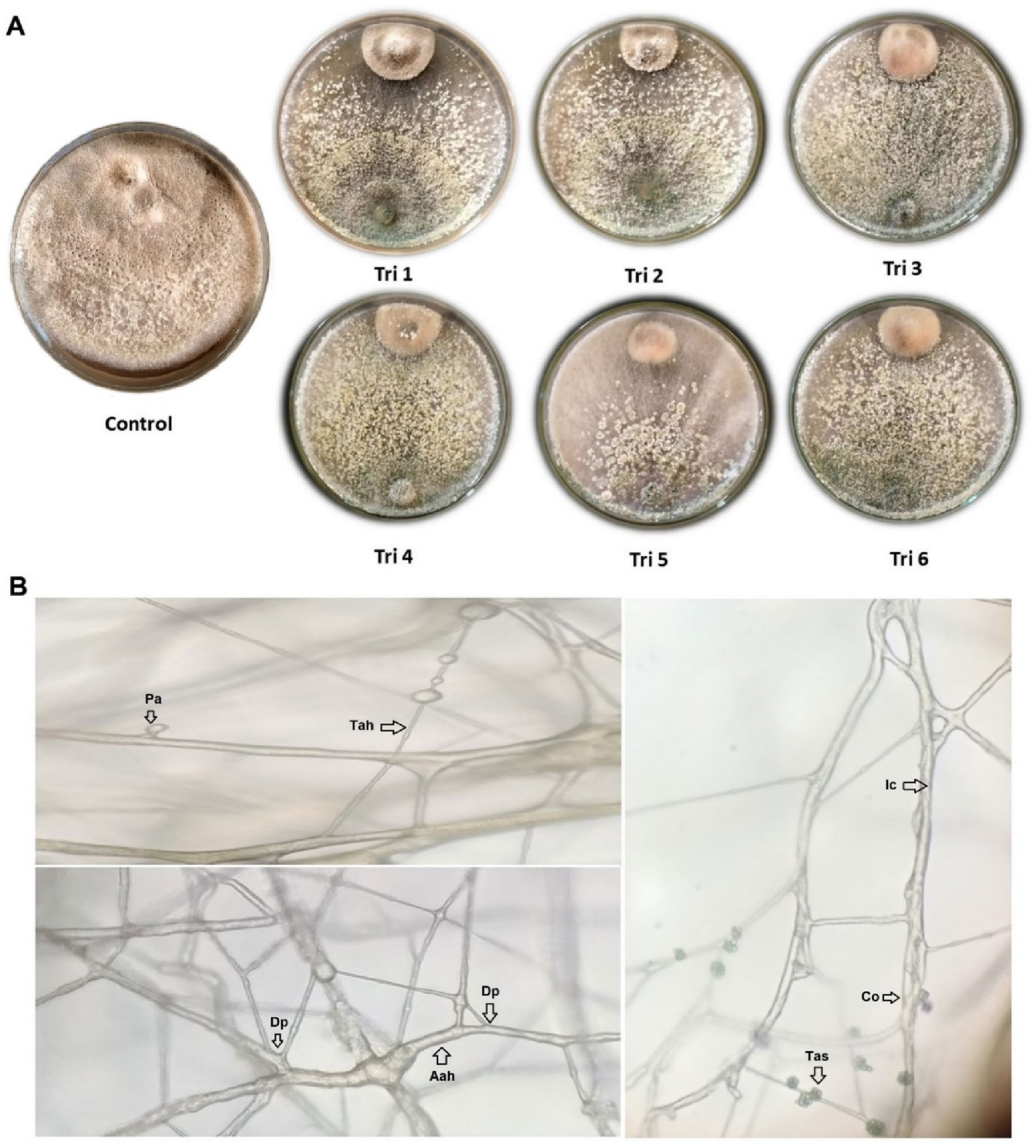
(**A**) Effect of *Trichoderma* isolates (Tri 1–Tri 6) against *Alternaria alternata* growth compared with control under laboratory conditions. (**B**) Hyphal interactions between *T. afroharzianum* isolate TRI07 (Tri 1) and *A. alternata* isolate (Alt3) in dual plate confrontations under a light microscope at 40×; *Pa* TRI07 papilla, *Tah* TRI07 hyphae, *Dp* direct penetration, *Aah* Alt3 hyphae, *Ic* intercellular growth of TRI07 in the host cells, *Co* coiling of TRI07 mycelium, *Tas* TRI07 spores. Scale bar = 50 μm.

Six *Trichoderma* isolates were examined for their ability to inhibit *A. alternata* mycelium’s growth (Table 2, Fig. 4). Significantly, *T. afroharzianum* isolate TRI07 (Tri 1) was the most effective isolate to inhibit the pathogen (76.66%), followed by Tri 2 (75.18%), Tri 5 (74.44%), Tri 6 (73.33%), and Tri 3 (71.11%). Tri 4 had the least effect on *A. altrernata* (68.51%).

**Table 2:** The effectiveness of *Trichoderma* isolates on *A. alternata* growth using dual culture plates.

*Trichoderma* isolates code	Inhibition percentage
Tri 1	76.66^a^ ± 1.11*
Tri 2	75.18^ab^ ± 1.28
Tri 3	71.11^cd^ ± 1.11
Tri 4	68.51^d^ ± 2.31
Tri 5	74.44^ab^ ± 2.22
Tri 6	73.33^bc^ ± 1.11
Control	0.00^e^ ± 0.00

*Values are means ± standard deviation.

If there are distinct letters next to the data values for inhibition%, it means that the values were significantly different at *p*-value ≤ 0.05.

#### Ethyl acetate extract of *Trichoderma* against pathogen mycelial growth

**Figure 5:**
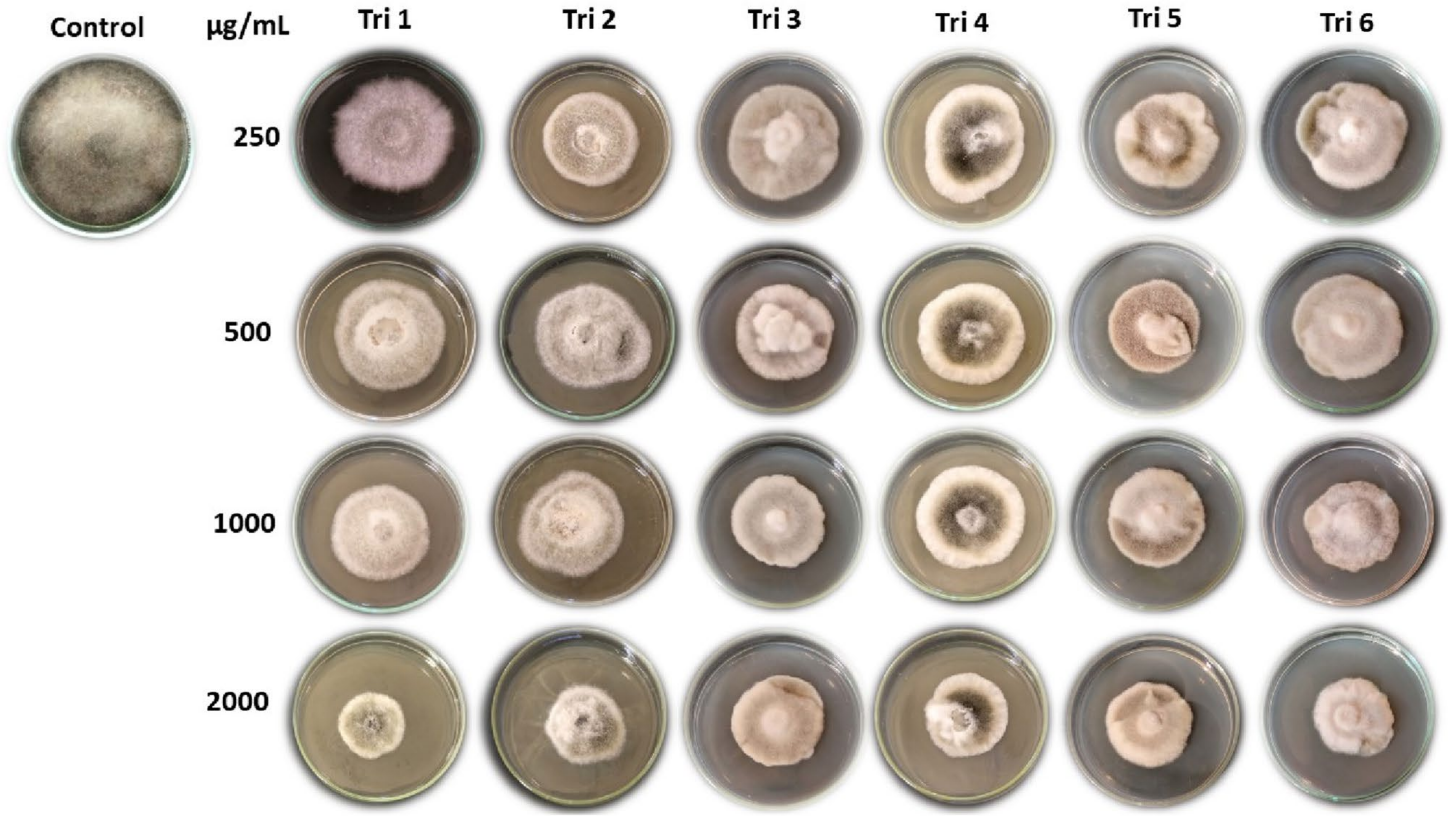
Effect of ethyl acetate extract of *Trichoderma* isolates (Tri 1–Tri 6) against *A. alternata* in vitro using different concentrations (250, 500, 1000, and 2000 µg/mL).

The data in Table 3 showed that the ethyl acetate extract of *Trichoderma* isolates reduced the growth of the *A. alternata* mycelium at concentration levels of 0, 250, 500, 1000, and 2000 µg/mL (Table 3, Fig. 5). The most suppressive bioagents against *A. alternata* isolate, with the highest significant inhibition growth were TRI07 (Tri 1), Tri 6, Tri 5, and Tri 2 with values of 38%, 34.96%, 33.77%, and 33.33%, respectively, while the least significant effect of inhibition percentage was 32.37% from Tri 3 and 30.88% from Tri 4.

**Table 3:** Effect of *Trichoderma* isolates (Tri 1–Tri 6) ethyl acetate extract against *A. alternata* isolate Alt3 growth in vitro.

*Trichoderma* isolates	Concentrations (µg/mL)					Mean of *Trichoderma* isolates
	0	250	500	1000	2000	
*T. afroharzianum* isolate TRI07 (Tri 1)	0.00 ± 0.00	38.51 ± 1.69*	42.59 ± 1.69	50 ± 1.11	58.88 ± 1.11	38^a^ ± 20.96
Tri 2	0.00 ± 0.00	35.92 ± 2.79	37.77 ± 1.92	39.62 ± 0.64	53.33 ± 1.92	33.33^cd^ ± 18.44
Tri 3	0.00 ± 0.00	35.92 ± 2.79	37.03 ± 1.69	43.70 ± 1.28	45.18 ± 1.28	32.37^d^ ± 17.22
Tri 4	0.00 ± 0.00	31.48 ± 3.20	34.44 ± 1.11	35.92 ± 1.69	52.59 ± 2.79	30.88^e^ ± 17.79
Tri 5	0.00 ± 0.00	36.66 ± 2.93	39.25 ± 0.64	44.44 ± 0.00	48.51 ± 1.69	33.77^bc^ ± 18.03
Tri 6	0.00 ± 0.00	33.70 ± 0.64	38.14 ± 4.20	48.88 ± 1.11	54.07 ± 1.69	34.96^b^ ± 19.68
Mean of concentrations	0.00^e^ ± 0.00	35.37^d^ ± 3.12	38.20^c^ ± 3.12	43.76^b^ ± 5.12	52.09^a^ ± 4.71	

LSD (*Trichoderma* isolates) at 0.05 = 1.29.

LSD (concentration) at 0.05 = 1.18.

LSD (Tri. *Con.) at 0.05 = 2.89.

*Values are means ± standard deviation.

#### *Trichoderma* isolates volatile compounds against *A. alternata*

**Figure 6:**
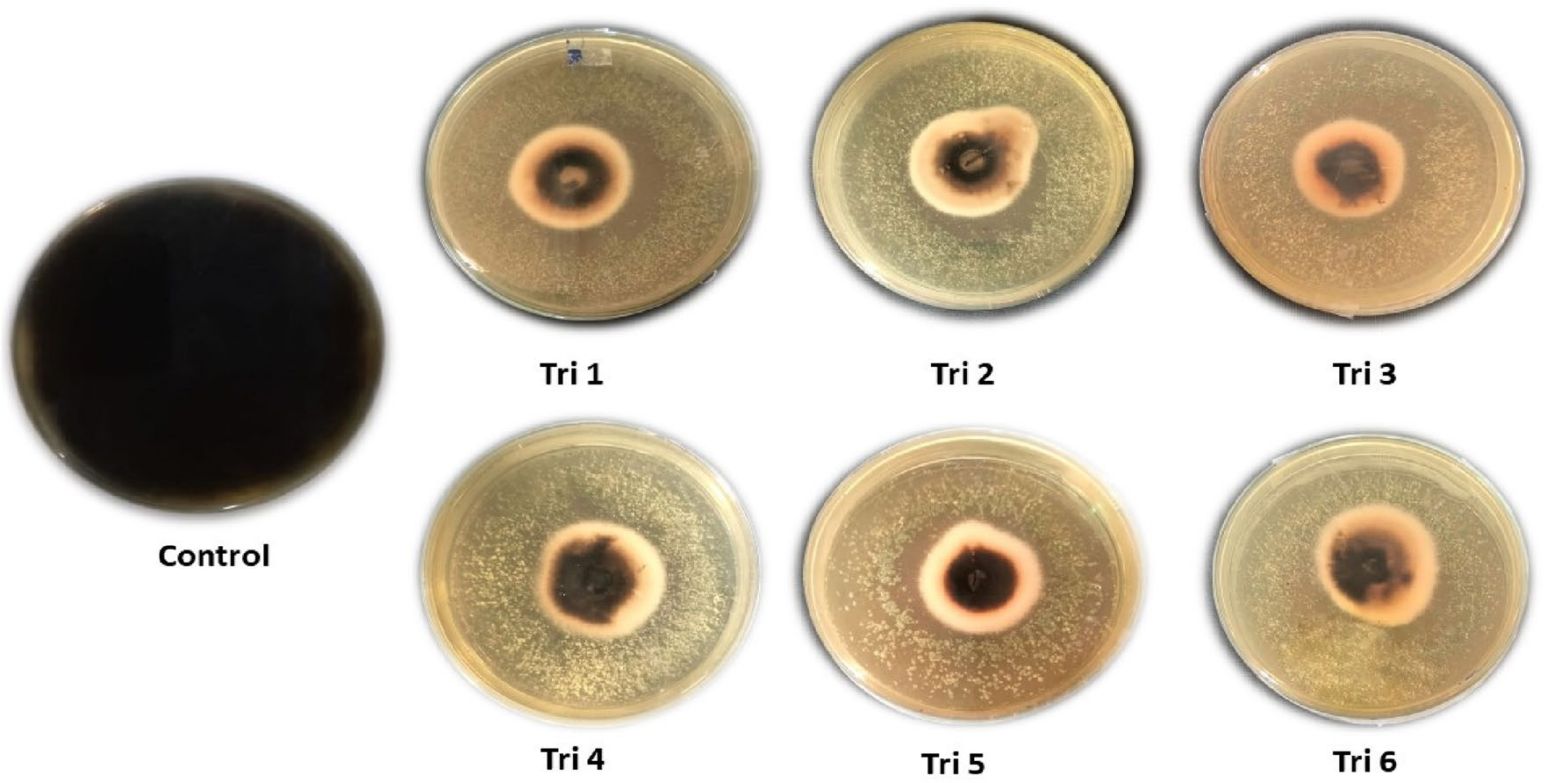
Effect of volatile organic compounds (VOCs) emitted from *Trichoderma* isolates (Tri 1–Tri 6) against *A. alternata* in vitro using the inverted sealed plate method.

In this study, the effect of volatile organic compounds (VOCs) generated from six *Trichoderma* isolates (Tri 1–Tri 6) against *A. alternata* isolate Alt3 growth was tested using the sealed plate method. The findings demonstrated that all *Trichoderma* isolates generated VOCs capable of inhibiting *A. alternata* growth (Table 4, Fig. 6). According to Table 4, *T. afroharzianum* isolate TRI07 (Tri 1) recorded the biggest proportion of VOCs that inhibited *A. alternata* growth (66.29%), followed by Tri 3 and Tri 5 with values of 65.55% and 64.07%, respectively. While Tri 4 and Tri 6 showed moderately significant effects on VOC emissions with values of 62.96% and 60.37%, respectively. On the other side, Tri 2 had the lowest significant inhibition percentage (60.00%).

**Table 4:** Effect of volatile organic compounds (VOCs) emitted from *Trichoderma* isolates against *A. alternata* growth in vitro.

*Trichoderma* isolates code	Inhibition%
*T. afroharzianum* isolate TRI07 (Tri 1)	66.29^a^ ± 0.64*
Tri 2	60.00^c^ ± 1.11
Tri 3	65.55^a^ ± 1.11
Tri 4	62.96^b^ ± 1.69
Tri 5	64.07^ab^ ± 0.64
Tri 6	60.37^c^ ± 2.31
Control	0.00^d^ ± 0.00

When the inhibition% data values are denoted by different letters, it means that the results differed significantly at *p*-value ≤ 0.05.

*Values are means ± standard deviation.

After conducting laboratory tests using various techniques, the results revealed that *T. afroharzianum* isolate TRI07 (Tri 1) is the most effective isolate for inhibiting *A. alternata* mycelium growth. Consequently, it was selected for further investigation under greenhouse conditions to assess its effects against the Alt3 isolate.

### GC–MS analysis

#### *Trichoderma afroharazianum*-ethyl acetate culture filtrate

Using GC–MS analysis, the bioactive components of the TRI07 isolate extract were identified. The identified components with their retention time (RT), compound names, peak areas (%), and match factor (MF) are presented in Table 5. The GC–MS analysis of the TRI07 extract’s ethyl acetate filtrate revealed the presence of 13 chemical constituents (Table 5, Fig. S2), and the most abundant of them were spathulenol (28.90%), triacetin (14.03%), aspartame (12.97%), oleic acid (6.39%), β-ionone (5.82%), 13-methylpentadecanoic acid methyl ester (5.63%), ethylene brassylate (4.42%), cyclobarbital (3.39%) and o-desmethylvinenlafaxine (3.58%).

**Table 5:** Identified compounds in ethyl acetate extract of *Trichoderma afroharzianum* TRI07 defined by GC–MS.

RT	Compound name	Area%	MF
18.69	Spathulenol	28.90	891
13.09	Triacetin	14.03	823
35.03	Aspartame	12.97	636
29.46	Oleic acid	6.39	697
16.40	*β*-Lonone	5.82	726
26.21	13-Methylpentadecanoic acid methyl ester	5.63	661
27.68	Ethylene brassylate	4.42	727
32.10	Strychane,1-acetyl-20à-hydroxy-16-methylene	4.04	645
20.56	2,3,4,5-Tetrahydroxypentanal	4.00	674
20.48	*o*-Desmethylvenlafaxine	3.58	656
5.65	Cyclobarbital	3.39	640
29.06	1-Tetradecanol	3.27	640
36.60	Digitoxin	1.85	672
4.03	Isophthalic acid	1.70	727

*RT* retention time, *MF* molecular formula.

### Analysis of VOCs from *T. afroharazianum* TRI07 by solid phase microextraction method (SPME)

From *T. afroharazianum* TRI07 pure culture, a total of 15 VOCs were found within the range of the measured masses and 34 VOCs of *T. afroharazianum* against *A. alternata* (Alt3) treatment (Tables 6, 7). The total number and amount of VOCs produced by TRI07 pure culture and its bioactivity against Alt3 were directly correlated. When comparing the total amount of VOCs from TRI07 pure culture and its bioactivity against Alt3, it was found that the presence of a few similar compounds in different amounts, such as ethanol, was the most abundant compound in the VOC profile released from TRI07 pure culture with a value of 49.51%, whereas in a dramatic way, the ethanol VOC content decreased in TRI07 against Alt3 treatment with a value of 24.29%. Also, propanoic acid, 2-methyl-, ethyl-**,** oxime-, methoxy-phenyl, 3-octanone, hydroperoxide**,** and 1-methylhexyl VOCs showed slightly higher rates in TRI07 pure culture with values of 0.51%, 5.39%, 8.53%, and 10.33%, respectively, than TRI07 against Alt3 VOCs with displayed values of 0.28%, 0.48%, 6.88%, and 1.06%, respectively.

**Table 6:** Volatile organic compounds (VOCs) produced from *T. afroharazianum* TRI07 by solid phase microextraction method (SPME).

Peak	RT	Name	Formula	Area %
1	0.86	Ethanol	C_2_H_6_O	49.51
2	1.073	1,3-Butanediol	C_4_H_10_O_2_	5.6
3	1.156	Hydroperoxide,1-methylhexyl	C_7_H_16_O_2_	10.33
4	1.257	Pentanal	C_5_H_10_O	1
5	1.916	Propanoic acid, 2-methyl-, ethyl ester	C_6_H_12_O_2_	0.51
6	4.545	Oxime-, methoxy-phenyl	C_8_H_9_NO_2_	5.39
7	5.756	1-Octen-3-one	C_8_H_14_O	10.82
8	5.917	3-Octanone	C_8_H_16_O	8.53
9	6.937	2,4,6-Cycloheptatrien-1-one, 4-methyl-	C_8_H_8_O	0.9
10	13.11	2H-Pyran-2-one, 6-pentyl	C_10_H_14_O_2_	3.52
11	16.606	Tetradecanoic acid	C_14_H_28_O_2_	0.69
12	19.23	*n*-Hexadecanoic acid	C_16_H_32_O_2_	0.62
13	21.556	Oxalic acid, cyclohexylmethyl ethyl ester	C_11_H_18_O_4_	1.1
14	26.696	Hexanedioic acid, bis(2-ethylhexyl) ester	C_22_H_42_O_4_	0.9
15	28.382	Oxalic acid, diallyl ester	C_8_H_10_O_4_	0.57

*RT* retention time.

**Table 7:** Volatile organic compounds (VOCs) produced from interaction between *T. afroharazianum* TRI07 and *A. alternata* Alt3 by solid phase microextraction method.

Peak	RT	Name	Formula	Area sum %
1	0.837	Ethanol	C_2_H_6_O	24.29
2	1.097	Hydroperoxide, 1-methylhexyl	C_7_H_16_O_2_	1.06
3	1.343	Acetamide, 2-fluoro-	C_2_H_4_FNO	1.13
4	1.672	1-Butanol, 3-methyl-	C_5_H_12_O	0.56
5	1.882	Propanoic acid, 2-methyl-, ethyl ester	C_6_H_12_O_2_	0.28
6	4.33	Oxime-, methoxy-phenyl	C_8_H_9_NO_2_	0.48
7	5.074	1-Nonanol	C_9_H_20_O	0.25
8	5.78	1-Octen-3-one	C_8_H_14_O	25.1
9	5.928	3-Octanone	C_8_H_16_O	6.88
10	7.129	Decanal	C_10_H_20_O	2.61
11	7.375	2-Octen-1-ol, (Z)-	C_8_H_16_O	0.67
12	11.281	Triacetin	C_9_H_14_O_6_	1.64
13	12.766	5,9-Undecadien-2-one, 6,10-dimethyl-, (E)-	C_13_H_22_O	0.41
14	13.13	2H-Pyran-2-one, 6-pentyl-	C_10_H_14_O_2_	4.69
15	13.403	7-epi-trans-sesquisabinene hydrate	C_15_H_26_O	0.26
16	13.557	9-Octadecenoic acid (Z)-, phenylmethyl ester	C_25_H_40_O_2_	0.42
17	14.7	Tetradecane, 2,6,10-trimethyl-	C_17_H_36_	0.96
18	14.777	*tert*-Hexadecanethiol	C_16_H_34_S	0.26
19	15.16	Formic acid, 3,7,11-trimethyl-1,6,10-dodecatrien-3-yl ester	C_16_H_26_O_2_	0.29
20	15.8	3-Chloropropionic acid, heptadecyl ester	C_20_H_39_ClO_2_	0.76
21	16.013	1-Hexadecanol, 2-methyl-	C_17_H_36_O	0.99
22	16.369	1-Dodecanol, 2-hexyl-	C_18_H_38_O	0.31
23	16.601	Tetradecanoic acid	C_14_H_28_O_2_	0.69
24	17.561	2-Dodecen-1-yl(-)succinic anhydride	C_16_H_26_O_3_	0.25
25	17.826	Pentadecanoic acid	C_15_H_30_O_2_	0.34
26	18.737	Hexadecanoic acid, methyl ester	C_17_H_34_O_2_	0.47
27	18.945	Hexadecenoic acid, Z-11-	C_16_H_30_O_2_	0.57
28	19.323	*n*-Hexadecanoic acid	C_16_H_32_O_2_	2.61
29	21.361	9-Octadecynoic acid, methyl ester	C_19_H_34_O_2_	0.29
30	21.448	9-Octadecenoic acid (Z)-, methyl ester	C_19_H_36_O_2_	0.61
31	21.579	9,12-Octadecadienoyl chloride, (Z,Z)-	C_18_H_31_ClO	0.28
32	22.074	*cis*-Vaccenic acid	C_18_H_34_O2	0.27
33	22.507	Octadecanoic acid	C_18_H_36_O_2_	0.51
34	26.793	Hexanedioic acid, bis(2-ethylhexyl) ester	C_22_H_42_O_4_	18.82

*RT* retention time.

**Figure 7:**
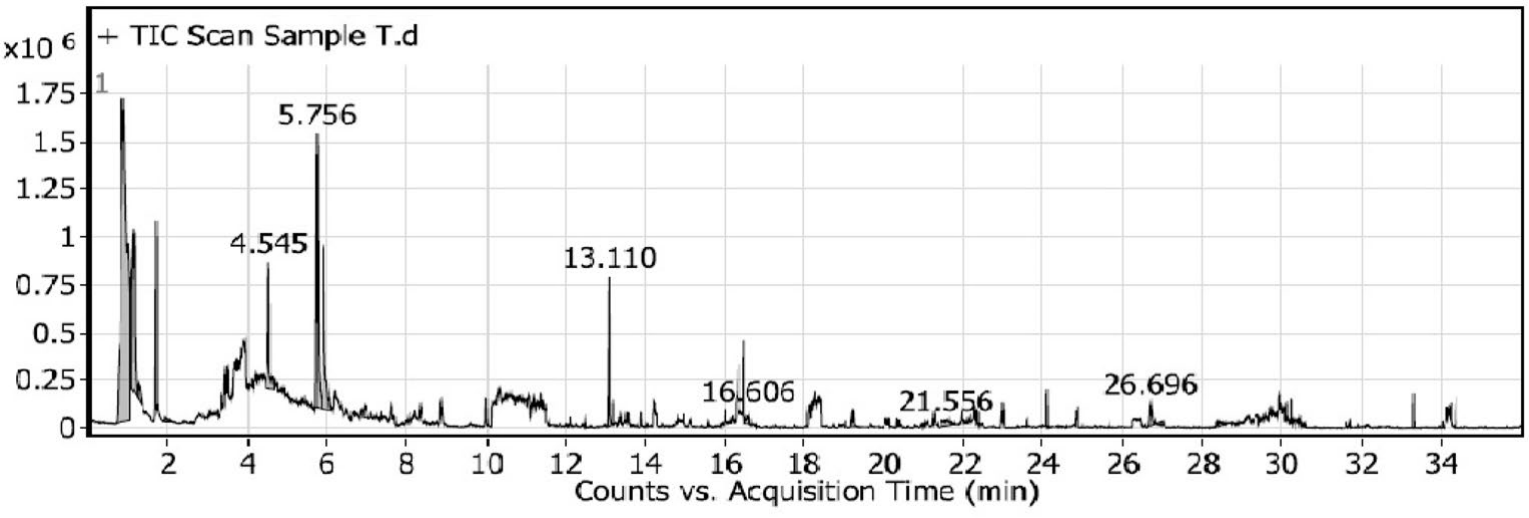
Volatile organic compounds (VOCs) produced by *T. afroharazianum* TRI07 by solid phase microextraction method.

**Figure 8:**
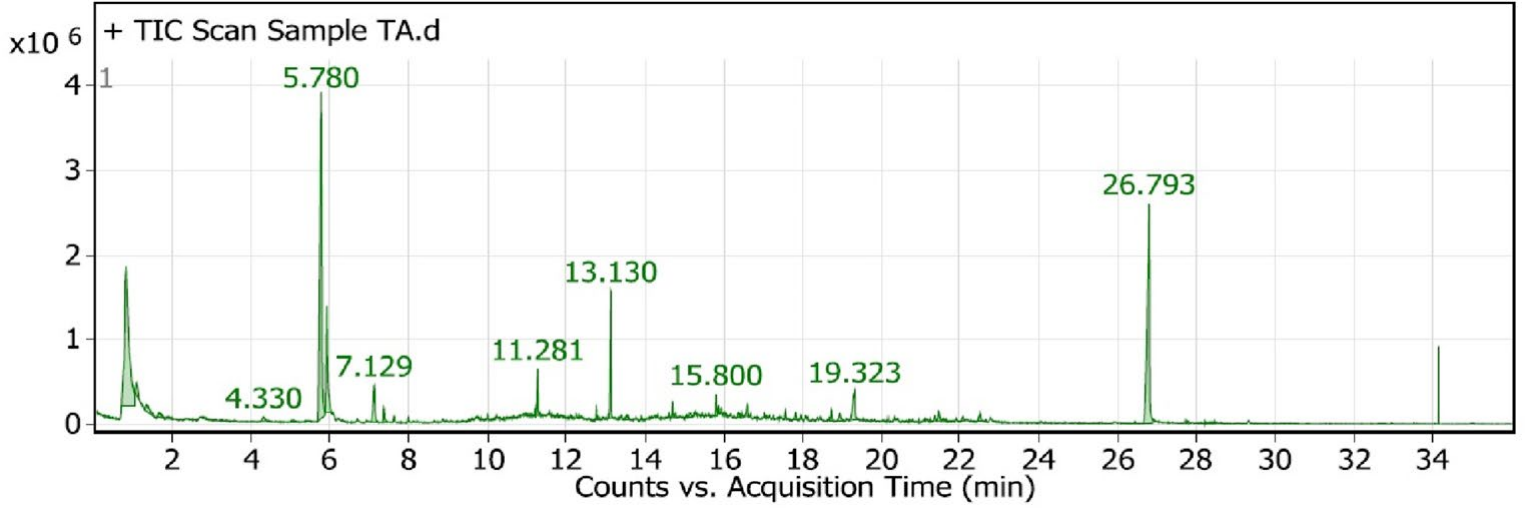
Volatile organic compounds (VOCs) emitted from the interaction between *T. afroharazianum* TRI07 and *A*. *alternata* Alt3 conducted by solid phase microextraction method.

In addition, some VOC values increased in the presence of the isolated pathogen Alt3 rather than in TRI07 pure culture, such as 1-octen-3-one (25.1%), 2h-pyran-2-one, 6-pentyl- (4.69%), n-hexadecanoic acid (2.61%), and hexanedioic acid, bis(2-ethylhexyl) ester (18.82%). Finally, tetradecanoic acid gave the same percentage (0.69%) in both VOC treatments (Figs. 7, 8).

### Greenhouse studies

#### Disease severity and incidence of *A. alternata* leaf spot on tomato plants

The disease severity (DS) and disease incidence (DI) of *Alternaria alternata* isolate Alt3 were recorded 30 days after transplanting seedlings. The obtained results (Table 8) showed that application of *T. afroharzinum* TRI07 reduced disease severity significantly compared with other treatments.

**Table 8:** Effect of *Trichoderma* isolates on *A. alternata* disease severity (DS%) and disease incidence (DI%).

Treatments	Disease severity (DS%)	Disease incidence (DI%)
A1	*0.00^c^ ± 0.00	0.00^c^ ± 0.00
A2	80.55^a^ ± 12.72	100^a^ ± 0.00
A3	16.66^bc^ ± 14.43	44.44^b^ ± 38.48
A4	38.88^b^ ± 12.72	100^a^ ± 0.00
A5	16.66^bc^ ± 14.43	44.44^b^ ± 38.48
A6	33.33^b^ ± 14.43	100^a^ ± 0.00
LSD value	22.37	39.52

*A1* tomato plant control, *A2* plants inoculated by *A. alternata* (Alt3) only, *A3* plants inoculated with *T. afroharzinum* TRI07 48 h before inoculation with Alt3, *A4* plants inoculated with TRI07 after 48 h inoculation with Alt3, *A5* plants treated with fungicide 48 h before inoculation with Alt3, *A6* plants treated with fungicide after 48 h of inoculation with Alt3.

*Values are means ± standard deviation. The presence of different letters next to the data values in each column indicates that the differences were significant at *p*-value ≤ 0.05.

Plants treated with TRI07 48 h before inoculation with Alt3 (A3) and plants treated with fungicide 48 h before inoculation with Alt3 (A5) showed the lowest level of disease severity (16.66%) with tomato leaf spot, followed by tomato plants treated with TRI07 after inoculation with Alt3 and tomato plants treated with fungicide 48 h after inoculation with Alt3 (A4, 38.88%, and A6, 33.33%, respectively). While the highest DS% was recorded on tomato plants inoculated by Alt3 only (A2, 80.55%) compared to the control plants (A1). On the other hand, A3 and A5 treatments showed the lowest value of disease incidence (44.44%). The application of A4 and A6 treatments showed the highest value of disease incidence (100%) compared to A2 treatment.

#### Effect of *Trichoderma afroharzianum* on tomato growth parameters

According to the results of the experiment conducted in a greenhouse, tomato plants in A3 treatment showed a substantial improvement (*p*-value ≤ 0.05) in their growth indices. The A3 treatment also significantly affected the lengths of the shoot and roots, which were measured at 71.55 cm and 32.22 cm, respectively. Tomato plants in A1 and A5 treatments had shoot and root lengths of 70.88, 30.77, 69.55, and 26.44 cm, respectively.

On the other hand, A4 and A6 treatments presented shoot and root lengths with values of (57.77 and 27.22 cm) and (63.00 and 24.66 cm), respectively. Meanwhile A3 increased the fresh and dry weight of the shoot system (13.37 and 4.16 g, respectively) compared with the control (A1) (13.51 and 4.01 g, respectively). Tomato fresh and dry shoot weight showed a high response to A5 and A6 treatments (12.45–4.03 g and 12.20–3.55 g, respectively). While A2 treatment showed the lowest fresh and dry weights of the tomato shoot system (11.27 and 3.25g, respectively).

For fresh and dry weight of tomato root (Table 9), plants in A3 treatment showed a highly significant response (11.71 and 1.98 g, respectively), and A5 treatment (11.64 and 1.84g, respectively) compared to control (A1). Furthermore, the lowest values were reported with A2 treatment (8.73 and 1.17 g, respectively). As compared to control (A1), the A3 treatment significantly improved the chlorophyll content (25.46 SPAD units), followed by the A5 and A6 treatments, with SPAD units of 22.12 and 20.55, respectively (Table 9). A4 treatment, with a 19.9 SPAD value, demonstrated moderate significance, while A2 treatment demonstrated the lowest significance (10.5 SPAD value).

**Table 9:** Vegetative parameters and chlorophyll content of tomato plants in vivo.

Treatments	Fresh weight (g)		Dry weight (g)		Length		Chlorophyll content (SPAD unit)
	Shoots	Roots	Shoots	Roots	Shoots	Roots	
A1	13.51^a^ ± 3.38*	11.01^ab^ ± 0.51	4.01^a^ ± 0.42	1.73^ab^ ± 0.36	70.88^a^ ± 4.80	30.77^a^ ± 1.30	21.49^b^ ± 1.78
A2	11.27^b^ ± 1.65	8.73^c^ ± 1.27	3.25^c^ ± 0.21	1.17^c^ ± 0.13	48.11^d^ ± 1.69	22.33^d^ ± 2.91	10.5^c^ ± 4.11
A3	13.37^a^ ± 1.54	11.71^a^ ± 0.65	4.16^a^ ± 0.22	1.98^a^ ± 0.25	71.55^a^ ± 1.50	32.22^a^ ± 1.98	25.46^a^ ± 3.63
A4	11.80^ab^ ± 1.64	9.60^bc^ ± 1.87	3.60^b^ ± 0.42	1.17^c^ ± 0.15	57.77^c^ ± 2.86	27.22^b^ ± 2.72	19.9^b^ ± 3.32
A5	12.45^ab^ ± 1.86	11.64^a^ ± 3.63	4.03^a^ ± 0.18	1.84^a^ ± 0.39	69.55^a^ ± 1.50	26.44^bc^ ± 2.78	22.12^b^ ± 1.96
A6	12.20^ab^ ± 2.36	10.85^ab^ ± 2.83	3.55^b^ ± 0.27	1.48^b^ ± 0.37	63.00^b^ ± 2.95	24.66^cd^ ± 3.70	20.55^b^ ± 1.69
LSD value	2.06	2.01	0.29	0.28	2.66	2.54	2.79

*A1* tomato plant control, *A2* plants inoculated by *A. alternata* (Alt3) only, *A3* plants inoculated with *T. afroharzinum* TRI07 48 h before inoculation with Alt3, *A4* plants inoculated with TRI07 after 48 h inoculation with Alt3, *A5* plants treated with fungicide 48 h before inoculation with Alt3, and *A6* plants treated with fungicide after 48 h of inoculation with Alt3.

The presence of different letters next to the data values in each column indicates that the differences were significant at *p*-value ≤ 0.05.

*Values are means ± standard deviation.

#### Antioxidant enzymes activities

Four antioxidant enzymes, namely catalase (CAT), peroxidase (POD), superoxide dismutase (SOD), and polyphenol oxidase (PPO), were distinguished upon *A. alternata* infection, *Trichoderma,* and fungicide treatments (Table 10). When compared to the control plant (A1), the *A. alternata* treatment (A2) had the greatest CAT activity value (0.15 µM/g FW), followed by the A3 treatment (0.089 µM/g FW). Contrarily, A4 treatment reduced CAT activity (0.074 µM/g FW) compared to control, which was then followed by A5 and A6 treatments with values of 0.034 and 0.05 µM/g FW, respectively. In terms of POD activity, the A3 treatment value was 1.24 µM/g FW, followed by the A2 treatment value of 1.04 µM/g FW, as opposed to the control (A1) with a value of 0.56 µM/g FW. The lowest value was shown with A5, 0.28, and A6, 0.31 µM/g FW, while the moderate value was obtained from the A4 treatment value of 0.51 µM/g FW.

**Table 10:** The activities of CAT, POD, SOD, PPO, MDA and H_2_O_2_ enzymes, total protein and total phenolic compounds (TPC) in tomato plant leaves under greenhouse study.

Treatments	CAT (µM/g FW)	POD (µM/g FW)	SOD (µM/g FW)	PPO (µM/g FW)	MDA (µM/g FW)	H_2_O_2_ (µM/g FW)	Protein (µg/mL)	TPC (mg GAE/g)
A1	0.089^b^ ± 0.003*	0.56^c^ ± 0.002	0.31^e^ ± 0.007	0.33^e^ ± 0.003	0.48^e^ ± 0.002	13.99^c^ ± 0.20	332.68^a^ ± 0.00	79.12^f^ ± 0.00
A2	0.15^a^ ± 0.002	1.04^b^ ± 0.0075	0.37^d^ ± 0.004	0.56^b^ ± 0.011	0.51^d^ ± 0.002	46.63^a^ ± 0.20	326.71^c^ ± 0.60	296.41^a^ ± 0.72
A3	0.089^b^ ± 0.004	1.24^a^ ± 0.011	0.51^a^ ± 0.002	0.46^c^ ± 0.002	0.53^c^ ± 0.003	37.70^b^ ± 0.00	333.56^a^ ± 3.98	176.83^e^ ± 0.72
A4	0.074^c^ ± 0.011	0.51^d^ ± 0.00	0.44^b^ ± 0.008	0.43^d^ ± 0.013	0.46^f^ ± 0.005	4.66^f^ ± 0.00	326.71^c^ ± 0.60	185.58^d^ ± 0.72
A5	0.034^e^ ± 0.004	0.28^f^ ± 0.011	0.37^d^ ± 0.008	0.45^d^ ± 0.041	0.66^a^ ± 0.004	6.63^d^ ± 0.20	329.52^b^ ± 0.00	203.70^b^ ± 0.72
A6	0.05^d^ ± 0.002	0.31^e^ ± 0.003	0.38^c^ ± 0.003	0.75^a^ ± 0.001	0.61^b^ ± 0.025	4.95^e^ ± 0.20	316.36^d^ ± 0.00	189.75^c^ ± 1.25

*A1* tomato plant control, *A2* plants inoculated by *A. alternata* (Alt3) only, *A3* plants inoculated with *T. afroharzinum* TRI07 48 h before inoculation with Alt3, *A4* plants inoculated with TRI07 after 48 h inoculation with Alt3, *A5* plants treated with fungicide 48 h before inoculation with Alt3, and *A6* plants treated with fungicide after 48 h of inoculation with Alt3.

The presence of different letters next to the data values in each column indicates that the differences were significant at *p*-value ≤ 0.05.

*Values are means ± standard deviation.

The tomato plants in A3 treatment exhibited the highest value of SOD activity (0.51 µM/g FW), followed by A4 treatment, 0.44 µM/g FW. Additionally, tomato plants in A6 showed a small increase in SOD activity (0.38 µM/g FW) greater than tomato plants in A4 or the healthy control plant (A1), which was not treated with a fungicide. The best value of induced PPO activity was 0.75 µM/g FW in A6 compared to the *A. alternata* treatment (A2). Additionally, compared to the control (A1), the PPO activity in the A3, A5, and A4 treatments was somewhat higher at 0.46, 0.45, and 0.43 µM/g FW, respectively (Table 10).

#### Oxidative stress markers assay

The current findings on tomato plants demonstrate the importance of H_2_O_2_ and MDA as indicators of oxidative stress. In the MDA results, the treatments (A5) and (A6) were 0.66 and 0.61 µM/g FW, compared to the healthy tomato plants (A1, 0.48 µM/g FW). While A4 displayed a low value (0.46 µM/g FW) compared to the control, A3 displayed a slightly higher value (0.53 µM/g FW) in MDA content compared to the A2 treatment. At the same time, the H_2_O_2_ assay revealed that A2 treatment had the maximum amount (46.63 µM/g FW) in plants, followed by A3 treatment (37.70 µM/g FW). Also, A4, A5, and A6 treatments encouraged a drop in H_2_O_2_ concentration with values of 4.66, 6.63, and 4.95 µM/g FW, respectively (Table 10).

#### Total protein assay

Tomato plants in A3 treatment showed the highest protein content, reaching 333.56 µg/mL, followed by A1 and A5 treatments (332.68 and 329.52 µg/mL, respectively). Furthermore, there was no significant difference between tomato plants in the A4 treatment (326.71 µg/mL) and the A2 treatment (326.71 µg/mL). On the other hand, A6 plants showed a slight increase in the total protein content of leaves (316.36 µg/mL) as shown in Table 10.

#### Total phenolic content

Total phenol content (TPC), expressed as mg GAE/g of tomato extract, was shown to accumulate in plants at high levels in the A2 (296.41 mg GAE/g), A5 (203.70 mg GAE/g), and T6 (189.75 mg GAE/g) treatments compared to the control A1 (79.12 mg GAE/g). While the A4 and A3 treatments accumulated fewer TPCs (185.58 and 176.83 mg GAE/g, respectively) (Table 10).

#### Phytochemicals in tomato leaf extract

Table 11 and Fig. S3 show the ethanolic extract HPLC chromatograms for the six different tomato plant treatments. Based on the HPLC results, the different treatments A1, A2, A3, A4, A5, and A6 had different amounts of polyphenolic compounds: 2770.72, 6049.75, 7953.78, 3468.25, 2250.69, and 1764.00 µg/g, respectively. It can be seen that the highest concentration of polyphenolic compounds (µg/g) in A1 were rutin (2555.52) and chlorogenic acid (57.30), in A2 were rutin (4855.79), chlorogenic acid (280.27), and gallic acid (232.23), in A3 were rutin (6423.57), chlorogenic acid (417.48), gallic acid (252.79), and caffeic acid (234.96), in A4 were rutin (2614.73), caffeic acid (160.80), and chlorogenic acid (159.06), in A5 were rutin (1564.34), chlorogenic acid (162.33), and caffeic acid (122.88), and in A6 were rutin (1317.2), chlorogenic acid (76.62), naringenin (60.91), and gallic acid (54.14).

**Table 11:** Detected polyphenolic compounds using HPLC analysis in tomato leaf extracts.

Compounds name	Concentration (µg/g)					
	A1	A2	A3	A4	A5	A6
Gallic acid	8.45	232.23	252.79	70.88	103.45	54.14
Chlorogenic acid	57.30	280.27	417.48	159.06	162.33	76.62
Catechin	6.46	46.16	50.59	28.96	26.66	24.84
Methyl gallate	2.54	28.97	46.81	15.69	19.48	19.37
Caffeic acid	11.84	170.99	234.96	160.80	122.88	47.46
Syringic acid	6.65	17.73	23.05	ND	ND	ND
Pyrocatechol	16.06	81.44	66.92	68.28	60.43	35.19
Rutin	2555.52	4855.79	6423.57	2614.73	1564.34	1317.2
Ellagic acid	33.58	68.55	71.6	126.62	30.46	23.64
Coumaric acid	0.88	19.25	19.47	13.17	6.77	4.08
Vanillin	3.59	60.66	65.77	33.45	25.86	22.31
Ferulic acid	15.97	64.06	38.93	76.25	49.24	55.94
Naringenin	26.39	95.39	75.56	74.12	59.45	60.91
Daidzein	2.32	4.85	5.08	4.39	4.01	3.42
Quercetin	13.24	21.18	13.28	7.97	13.67	17.89
Cinnamic acid	2.5	2.23	1.61	2.73	1.66	0.99
Apigenin	ND	ND	ND	11.15	ND	ND
Kaempferol	ND	ND	70.68	ND	ND	ND
Hesperetin	7.43	ND	75.63	ND	ND	ND
Total	2770.72	6049.75	7953.78	3468.25	2250.69	1764.00

*A1* tomato plant control, *A2* plants inoculated by *A. alternata* (Alt3) only, *A3* plants inoculated with *T. afroharzinum* TRI07 48 h before inoculation with Alt3, *A4* plants inoculated with TRI07 after 48 h inoculation with Alt3, *A5* plants treated with fungicide 48 h before inoculation with Alt3, and *A6* plants treated with fungicide after 48 h of inoculation with Alt3, *ND* not detected.

Other compounds like naringenin and quercetin as flavonoid compounds (µg/g) were overexpressed in A2 plants with an accumulation value of 95.39 and 21.18, respectively, compared to A1 plants (26.39 and 13.24). In addition to catechin, rutin and vanillin compounds were detected in the A3 treatment at high concentrations (50.59, 6423.57, and 65.77, respectively).

## Discussion

*Alternaria* leaf spot typically affects many vegetables, including tomato plants. One fungal isolate of the tomato leaf spot pathogen, identified as *Alternaria alternata* Alt3, was investigated. Using Simmons’ morphological characteristics, including colony morphology, size, conidial form, and septation pattern of conidia, the fungus strain was identified morphologically^79^. Based on ribosomal internal transcribed spacer (ITS) DNA sequence analysis, PCR was recently utilized to detect *Alternaria* spp. in tomato samples^43^. Therefore, depending on morphological characters and amplification and sequencing of ITS region, the pathogen was identified as *A. alternata*^80^.

According to the morphological characteristics of the *Trichoderma* isolates and genetical characterization, the most effective isolate was identified using the ITS, *tef*1, and *rpb*2 genetic markers as *Trichoderma afroharzianum* isolate TRI07. Within *Trichoderma* species complexes, it has been demonstrated that ITS sequence analysis is poor at separating closely related species^81^. *Tef*1 and *rpb*2 gene sequences are particularly instructive and have been demonstrated to be helpful in examining closely related strains at the species level^82,83^ and popular markers for classifying *Trichoderma* strains^84^.

Chemical fungicides are an efficient way to control the pathogen *A. alternata*. However, it might not always be appropriate to use fungicides to manage fungal infections in plants. Due to the hazards involved, chemical control of plant diseases is waning in popularity^85^. However, biocontrol agents are an environmentally friendly and sustainable replacement for dangerous fungicides^86^.

*Trichoderma* species are considered one of the most significant biological pest controllers because they reduce plant diseases through a variety of methods, including competition, mycoparasitism, antibiosis, and the induction of systemic resistance^87^. One of the most effective *Trichoderma* fungi for use as plant disease-suppressing inoculants is *T. afroharzianum*^88^.

In our in vitro studies we used three techniques to test the inhibition for mycelial growth of *A. alternata* like dual culture technique, food poison technique and volatile organic compounds (VOCs) which showed that *T. afroharzianum* was the most effective isolate to inhibit *A. alternata* fungus. In the initial dual culture assessments conducted in this investigation, *T. afroharzianum* TRI07 exhibited a growth rate three times higher than that of the pathogen. This superior growth rate provides TRI07 isolate with a significant competitive advantage for space and nutrients compared to *A. alternata*. During dual in vitro confrontations, TRI07 isolate not only suppressed the growth of the pathogens but also engaged in mycoparasitism against the pathogen. This mycoparasitic mechanism was corroborated through microscopic images of the confrontation assays. Microscopic examinations indicated that *T. afroharzianum* deployed a multifaceted mechanism against the tested *A. alternata* pathogen. This strategy involved hyper-sporulation, conidial binding to various structures of pathogenic fungi, and swift dissemination within the microenvironment of pathogens. *Trichoderma* surrounded the pathogen’s hyphae, depriving them of nutrients. The engagement of lytic enzymes played a crucial role in this mechanism, inducing morphological alterations and lysis of the pathogen’s conidia, thereby promoting mycoparasitism. The intricate process of direct fungal attack (mycoparasitism) involves sequential events of recognition, attack, and subsequent penetration and killing of the host^89^. Similar outcomes were observed in studies involving *Trichoderma* strains, where their conidia enveloped those of *Botrytis cinerea*, inhibiting germination and subsequent pathogen development, and morphological changes were also documented in *Fusarium* sp.^90,91^. The attack on pathogenic fungi involving conidia and coiling hyphae could be prompted by organic compounds and/or nutrient gradients emanating from the pathogens and sensed by *Trichoderma*^92^. Meanwhile, *Trichoderma harzianum* and *T. viride* strains performed best in a dual culture experiment against *A. alternata* with inhibition percentages of 75.04 and 67.83%, respectively^93^. Additionally, the hyphal penetration of *T. harzianum* into the hyphae of *A. alternata*, *F. proliferatum* and *B. sorokiniana* was demonstrated to be another mycoparasitic mechanism of action^94,95^.

The current study revealed that the ethyl acetate extract of all *Trichoderma* species efficiently suppressed the growth of the pathogen. The *Trichoderma* species are recognized for their ability to generate several secondary metabolites. The literature supports similar conclusions, Murtaza et al.^96^ examined the antifungal properties of five *Trichoderma* species, specifically *T. viride*, *aureoviride*, *reesei*, *koningii*, and *harzianum*, against *Alternaria citri*. The culture filtrate of *T. harzianum* exhibited remarkable efficiency in suppressing the growth of the investigated fungal species, achieving a suppression rate of up to 93%. The fractionation-guided bioassay of *T. harzianum* metabolites revealed a significant 68% reduction in the growth of *A. citri* when exposed to a 1% concentration of ethyl acetate fraction.

The identification of bioactive constituents in the ethyl acetate extract of *T. afroharazianum* was conducted through the application of gas chromatography-mass spectrometry (GC–MS) analysis. This analytical technique was employed to detect volatile organic compounds (VOCs) and non-volatile organic compounds (non-VOCs) present in the culture filtrates of *T. atroviride* and *T. asperellum*, respectively^97^. The ethyl acetate analysis revealed several compounds present in the *T. afroharzianum*, with significant proportions represented by specific constituents. Among these, spathulenol was the predominant compound, constituting 28.90% of the total area. Triacetin and aspartame followed, contributing 14.03% and 12.97%, respectively. Notably, oleic acid, β-lonone, and 13-methylpentadecanoic acid methyl ester were also identified as substantial components, each accounting for 6.39%, 5.82%, and 5.63% of the area, respectively. Moreover, ethylene brassylate, strychane, 1-acetyl-20à-hydroxy-16-methylene, 2,3,4,5-tetrahydroxypentanal, o-desmethylvenlafaxine, cyclobarbital, and 1-tetradecanol were detected in varying proportions, each contributing to the overall chemical composition of *T. afroharzianum* extract. The high percentage of spathulenol is noteworthy due to its reported biological activities, including antimicrobial properties. In other study, the primary compound identified in the essential oil of *Baccharis dracunculifolia* was spathulenol, an oxygenated sesquiterpene characterized by high hydrophobicity. The antimicrobial activity of sesquiterpenes, including spathulenol, is often attributed to a cell membrane-disrupting mechanism, leading to the release of K^+^ ions from pathogen cells, which increase the permeability across the plasma membrane, facilitating interaction with intracellular proteins and/or intra-organelle sites^98,99^. With a surface polarity of 20.2 Å^2^, spathulenol possesses one hydrogen bond donor and one acceptor^100^. According to Pajouhesh and Lenz^101^, compounds with a polar surface area (PSA) of 60 Å^2^ or less are fully absorbed by the cell, indicating the ability of spathulenol to traverse cellular membranes. The observed discrepancies in efficacy could perhaps be attributed to the presence of spathulenol, β-phellandrene, germacrene D, bicyclogermacrene, and 1,8-cineole in the specimens from Mpumalanga. These constituents have previously been identified as the primary chemicals in oils that exhibit antifungal properties^102,103^. Triacetin, one of the minor compounds, also has antifungal activity^104^. A study conducted to explore alternative strategies for addressing the presence of major plant disease pathogens, including *Pythium ultimum*, *Rhizoctonia solani*, *Crinipellis perniciosa*, and *Pyrenophora avenae* and their findings, as reported by Walters et al.^105^, suggest that linolenic, linoleic, and oleic acids could potentially offer beneficial effects in this regard. *Tychonema bourrellyi* culture filtrates contained a significant amount of β-ionone, which may be the source of the organism’s allelopathic biocidal activity^106^.

The results of our investigation indicate that *T. afroharazianum* emitted a total of 34 VOCs in response to *A. alternata* in liquid culture. Notably, the VOCs identified were 1-octen-3-one, 2H-pyran-2-one, 6-pentyl, *n*-hexadecanoic acid, and hexanedioic acid, bis (2-ethylhexyl) ester. These compounds exhibited significant antifungal properties when tested in vitro. The chemical known as acetophenone was shown to possess antifungal properties in laboratory conditions when tested against *Penicillium italicum*. This discovery was made among a group of VOCs produced by *T. afroharzianum* T22, as reported by Li et al.^107^. The VOCs produced by *T. harzianum* exhibited significant (p < 0.05) antifungal activity against *Botrytis cinerea*, *A. panax*, *Cylindrocarpon destructans*, and *Sclerotinia nivalis*. Joo et al.^108^ also conducted experiments to evaluate the effects of the treatment on plant growth promotion, repression, and enhancement.

A recent study conducted by Phoka et al.^109^ revealed that the endophytic fungus *T. asperelloides* PSU-P1 produces notable VOCs that contribute to its antifungal activities. Specifically, the identified VOCs were 2-methyl-1-butanol and 6-pentyl-2H-pyran-2-one. The development of *Colletotrichum gloeosporioides* and *A. alternata* is inhibited by the *T. koningiopsis* T2 strain through the production of metabolites derived from VOCs and non-VOCs^110^. The presence of bioactive chemicals in the extract suggests that the VOCs emitted by *T. harzianum* have a significant role as both an antioxidant and an antifungal agent, as indicated by Lakhdari et al.^111^.

In the greenhouse study, the highest DS% and DI% values were found in the A3 treatment in vivo tests with tomato plants treated with *T. afroharzianum* 48 h before inoculation with *A. alternata*. These findings are consistent with Chowdappa et al.^112^ who reported that *B. subtilis* and *T. harzianum* inhibited the growth of *P. infestans* and *A. solani *in vitro. As reported by Elsherbiny et al.^113^ *T. afroharzianum* significantly enhanced the growth of tomato plants, including plant height, number of leaves/plant, dry weight, and root activity, through colonization in the rhizosphere and root system. *T. afroharzianum* also increased tomato growth parameters. For instance, *T.* *afroharzianum* strain T22, the active ingredient of a commercial biofungicide product^114^ was found to enhance plant growth of significant horticultural crops, such as tomatoes, peppers, lettuce, ornamentals, and woody crops, and to control diseases under field and greenhouse conditions^115–117^. Through effective colonization in the plants’ rhizosphere and root system, *T. afroharzianum* TM2–4 considerably accelerated the growth of tomato plants in terms of dry weight, number of leaves per plant, plant height, and root activity^118^.

According to our study, *T. afroharizum* enhanced defense and detoxifying mechanisms, resulting in quick and effective responses to pathogen inoculation. Additionally, the activities of phenolics, flavonoids, total protein, CAT, SOD, and PPO may be crucial for tomato survival when under stress from fungi. Reactive oxygen species (ROS) are produced when a plant experiences stress and can affect plant growth by damaging DNA, proteins, and membrane systems^119^. The ability of enzymes like catalase and superoxide dismutase (SOD) to “clear out” ROS is reflected in their enzyme activity, which indirectly indicates the plant’s capacity to combat and reduce ROS^120^. High activities of POD and SOD were seen in a mutant soybean (*Glycine max*) that induces expression of the Tachi gene from *T. asperellum*. This mutant exhibited higher resistance to *S. sclerotiorum*^121^. Furthermore, *T. harzianum* inoculation markedly boosted the activity of POD, CAT, and PPO in squash^122^. Additionally, *T. afroharzianum* elevated CAT activity and decreased H_2_O_2_ content^123^. Secondary metabolites, such as vanillic, chlorogenic, and caffeine acids, were present in tomato leaves than in red fruits. The findings imply that inedible tomato plant components can be exploited as a source of raw materials for antioxidants, anti-inflammatory drugs, fungistats, and insecticides^124^. Trihydroxybenzoic acid, or gallic acid, is a naturally occurring polyphenol chemical that is present in a variety of plant species and has been proven to have antifungal and antibacterial activities^125^. In our recent work, we found that the utilization of *T. pubescens* either independently or in combination with other interventions for the management of plant pathogen infections led to elevated concentrations of phenolic acids, including chlorogenic and coumaric acids^57^.

In summary, treating plants with *T. afroharzianum* yielded positive outcomes in terms of antioxidant response and plant health. Enhanced production of antioxidant enzymes (CAT, POD, SOD, PPO) demonstrated improved defense mechanisms. However, infected plants showed increased MDA and H_2_O_2_ levels, indicating disease-related cellular damage. *T. afroharzianum* A3 treatment also led to higher levels of phenolics, known for their antioxidant properties. These findings suggest that *T. afroharzianum* has the potential to serve as an effective biocontrol agent against leaf spot disease, ultimately leading to enhanced post-crop productivity and improved plant health.

In light of recent reports indicating that *T. afroharzianum* has been associated with causing diseases in plants such as maize^126^, and there have been concerns about its potential impact on other plant cultivars, it is crucial to address the potential limitations and risks associated with the use of this biocontrol agent. While our study focuses on the positive aspects of *T. afroharzianum* in controlling *Alternaria* leaf spot in tomato, it is essential to recognize the need for further investigation into its potential effects. The comment raises valid concerns regarding the genetic diversity within the *T. afroharzianum* cluster, suggesting that variations in behavior among different strains may be attributed to genetic differences. This observation underscores existing knowledge gaps in understanding the genetic intricacies of *T. afroharzianum*. Consequently, it becomes imperative to conduct comprehensive genetic studies to unravel strain-specific characteristics and shed light on their varied pathogenic behaviors. Another critical aspect to consider is the influence of environmental factors and host-specific interactions. Regional climate variations and diverse plant cultivars may contribute to varying responses in *Trichoderma* behavior. Recognizing the multifaceted nature of these interactions is essential for interpreting contradictory findings in studies conducted in different regions and under different environmental conditions. The complexity of studying *Trichoderma* behavior necessitates a thorough consideration of methodological variations. Differences in experimental protocols between studies may contribute to conflicting outcomes. To address this, it is crucial to acknowledge these methodological nuances and advocate for standardized approaches. Comparative studies that align methodologies will be instrumental in validating and reconciling diverse research findings. The observed pathogenicity of certain *Trichoderma* strains raises questions about their application as biocontrol agents. Conducting a comprehensive risk–benefit analysis becomes paramount. While recognizing the potential benefits of *Trichoderma* in biocontrol, it is equally important to assess and communicate the associated risks. This cautious and well-informed approach is critical for determining the suitability of *Trichoderma* strains in agricultural practices.

Finally, future research and field trials should delve into the comprehensive assessment of *T. afroharzianum* safety and potential limitations in practical agricultural applications.

## Conclusions

Alternative environmentally acceptable bioagents are required to control plant diseases in the agriculture sector. Our research demonstrated the impact of *T. afroharzianum* on the in vitro and greenhouse-induced leaf spot disease caused by *A. alternata*. In the experimental greenhouse, *T. afroharzianum* displayed a highly significant increase in shoot and root length, shoot and root fresh and dry weight, and chlorophyll contents. The growth of *A. alternata *in vitro was greatly slowed down using dual culture techniques, volatile organic compounds, and *Trichoderma* extract. The GC–MS analysis for VOCs of *T. afroharzianum* with *A. alternata* showed increasing compounds like 1-octen-3-one, 2H-pyran-2-one, 6-pentyl, *n*-hexadecanoic acid and hexanedioic acid, bis (2-ethylhexyl) ester. *T. afroharzianum* treatment exhibited an increase in the synthesis of antioxidant enzymes (CAT, POD, SOD, and PPO) after 21 days of inoculation in vivo, and high MDA and H_2_O_2_ levels were found in the infected plants. The HPLC results of *T*. *afroharzianum* A3 treatment showed increasing in gallic, chlorogenic, caffeic and coumaric acids. To prevent the leaf spot disease and post crop productivity, *T. afroharzianum* causes systemic resistance, inhibits the growth of *A. alternata*, and fosters the development of tomato plants.

*Trichoderma afroharzianum* TRI07 stands out due to its potent antagonistic effects against various plant pathogens, showcasing a higher efficacy compared to other strains within the Trichoderma genus. Its distinct ability to enhance plant growth and induce systemic resistance makes it a promising candidate for biocontrol. Existing control methods often face limitations such as environmental concerns, resistance development, and inconsistent efficacy. TRI07’s unique features, including enhanced tolerance to environmental stressors, contribute to its potential as a sustainable alternative to conventional chemical approaches. This study innovatively explores TRI07’s molecular mechanisms, emphasizing its genetic attributes that contribute to heightened biocontrol capabilities. By dissecting these mechanisms, the research aims to develop more targeted and effective biocontrol strategies, overcoming the shortcomings of current methods. This study offers a sustainable strategy for suppressing *Alternaria* leaf spots, a significant pathogen affecting tomato crops. The approach relies on biological control and has the potential to be integrated with other environmentally friendly practices, serving as an alternative to chemical fungicides in various processes.

### Supplementary Information

Supplementary Information.

## Data Availability

The authors affirm that the article encompasses all the data generated and analyzed during this study. The raw sequence data for the ITS region, the translation elongation factor alpha 1 gene, and the RNA polymerase II subunit gene for all specimens were submitted to the NCBI GenBank database (https://www.ncbi.nlm.nih.gov/) under the accession numbers (OQ888806, OQ820171, OR125580 and OR125581).
