# Supplementary material for: Trichoderma afroharzianum TRI07 metabolites inhibit Alternaria alternata growth and induce tomato defense-related enzymes
Source: Sci Rep. 2024 Jan 22;14:1874. doi: 10.1038/s41598-024-52301-2 (PMC10803357; doi:10.1038/s41598-024-52301-2)
Supplement: Supplementary file 1 — Supplementary Information. [file 41598_2024_52301_MOESM1_ESM.docx]

**Supplementary file**

## ***Trichoderma afroharzianum* TRI07 metabolites inhibit *Alternaria alternata* growth and induce tomato defense-related enzymes**

**Bassant Philip^1,*^, Said I. Behiry^1,*^, Mohamed Z. M. Salem^2,*^, Mostafa A. Amer^1^, Ibrahim A. El-Samra^1^, Ahmed Abdelkhalek^3^, and Ahmed Heflish^1^**

^1^Agricultural Botany Department, Faculty of Agriculture (Saba Basha), Alexandria University, Alexandria 21531, Egypt; [bassantphilip3@alexu.edu.eg](mailto:bassantphilip3@alexu.edu.eg); [said.behiry@alexu.edu.eg](mailto:said.behiry@alexu.edu.eg), [maaamer@hotmail.com](mailto:maaamer@hotmail.com); [ibelsamra@hotmail.com](mailto:ibelsamra@hotmail.com); [ahmed_abdelbary@alexu.edu.eg](mailto:ahmed_abdelbary@alexu.edu.eg)

^2^ Forestry and Wood Technology Department, Faculty of Agriculture (El-Shatby), Alexandria University, Alexandria 21545, Egypt; [mohamed-salem@alexu.edu.eg](mailto:mohamed-salem@alexu.edu.eg)

^3^Plant Protection and Biomolecular Diagnosis Department, ALCRI, City of Scientific Research and Technological Applications, New Borg El Arab City 21934, Egypt; [aabdelkhalek@srtacity.sci.eg](mailto:aabdelkhalek@srtacity.sci.eg)

***Corresponding authors:** [bassantphilip3@alexu.edu.eg](mailto:bassantphilip3@alexu.edu.eg); [said.behiry@alexu.edu.eg](mailto:said.behiry@alexu.edu.eg); [mohamed-salem@alexu.edu.eg](mailto:mohamed-salem@alexu.edu.eg)

**Methods**

**Assay of peroxidase activity (POD)**

Utilizing pyrocatechol and hydrogen peroxide as substrates, the activity of POD was quantified. The enzymatic reaction was carried out by combining 150 mL of enzyme extract with 1500 μl of 0.1 M phosphate buffer (pH 6.8) that contained 30 mM pyrocatechol and 30 mM H_2_O_2_. The optical density of reaction mixture at 470 nm were determined every 20 sec ^1^ . A peroxidase activity unit was measured as the difference in absorbance/min, and its specific activity was measured as the number of enzyme units/mg of soluble protein ^2^.

**Assay of superoxide dismutase activity (SOD)**

A 3 mL of the reaction mixture and 100 µL of the enzyme extract were combined to begin the reaction. A 50 mM potassium phosphate buffer (pH 7.6), 13 mM methionine, 2 μM riboflavin, 0.1 mM EDTA, and 75 μM NBT were all present in the reaction mixture. The reaction mixture was used to create a blank, however, it was devoid of NBT and enzyme. The solution-filled tubes were exposed to 400 W bulbs for 15 minutes, and the absorbance was measured right away at 560 nm ^3^.

**Assay of catalase activity**

The CAT reaction mixture (3000 µL) contained of 50 mM phosphate buffer (pH 7.0), 15 mM H_2_O_2_ and 0.1 mL enzyme extract. The addition of the enzyme extract triggered the process. The reaction mixture's absorbance at 440 nm were recorded every 20 s. One unit of CAT activity was defined as an absorbance change of 0.01 unit /min ^4^. The specific activity of the enzyme was reported as units/mg protein.

**Assay of PPO activity**

By measuring the initial rate of product formation spectrophotometrically, PPO activity on pyrocatechol was assessed ^5^. In this test, 200 µL of the enzyme preparation was added to a reaction medium containing 30 mM pyrocatechol produced in 3.0 mL of 100 mM sodium phosphate buffer (prepared by mixing monosodium phosphate and di sodium phosphate, pH 6.8), and the mixture was then incubated at room temperature for 20 min. The absorbance was measured at 480 nm. 200 µL of a blank sample included an enzyme that had been pre-heated to 100 °C for 45 min in a boiling water bath and then quickly chilled on ice.

**Assay of lipid peroxidation (MDA)**

Lipid peroxidation was analyzed in plant tissues through measurement of malondialdehyde ^6^. Three fresh leaf samples totaling around 0.25 g were homogenized in 5 mL of 0.1% TCA and centrifuged for 15 min at 2500 rpm. A sample of 1 mL was combined with 4 mL of TBA (0.25%) and heated at 95 °C for 30 min before cooling in an ice bath for 10 min and centrifuging. The absorbance was measured at 532 nm and 600 nm in the supernatant as an indicator of the concentration of malondialdehyde and expressed as µM/g fresh weight (FW).

**Assay of hydrogen peroxide (H_2_O_2_)**

The KI method with a straightforward modification was used to quantify hydrogen peroxide (H2O2) in fresh leaf samples of tomato plants (Junglee et al., 2014). TCA 0.1% was used to smash fresh plant samples (100 mg), which was followed by centrifugation to obtain pure homogenate. By combining 1 mL of plant homogenate with 2 mL of KI solution (1 M KI in 10 mM phosphate buffer, pH 7.0), the H2O2 reactions were quantified. After 20 minutes, the absorbance was measured at 390 nm, and using the extinction coefficient of H2O2 (0.28/M/ cm), the findings were reported as µM/g FW.

**Estimation of total protein**

A colorimetric technique was used to assess the total protein concentration of the extract ^7^. By comparing the amount of Coomassie Brilliant Blue G-250 dye that binds to the unknown protein solution to known standards, we can calculate the protein concentration. 50 µL of extract was first mixed with 2.5 mL of Coomassie Blue in 0.15 M NaCl solution as a solvent, with 50 µL of 0.15 M NaCl serving as a blank. For 5 min, the reaction mixtures were left at room temperature. Standard solutions were made using bovine serum albumin at specific concentrations (0 –1 mg/mL). The standard curve was used to determine the protein concentration of the extract after reading the absorbance at 595 nm against a blank.

**Total Phenolic Content in Tomato Plants**

The total phenolic content (TPCs) of tomato leaf samples obtained from all treatments was calculated using the Folin-Ciocalteu method ^8^. To get a final concentration of 1 mg/mL, 10 mL of 95% ethanol was used to liquefy 0.1 g of leaf extract. 750 µL of the FC reagent and 100 µL of the extract were combined. For five minutes, the liquid composite was kept at 25 C. The mixture was then added to 750 µL of Na_2_CO_3_, and the tube was gently shaken to blend it. At 725 nm, the mixture was measured after an hour of incubation. A calibration curve was used to calculate the TPC using gallic acid equivalent (mg GAE/g of extract).


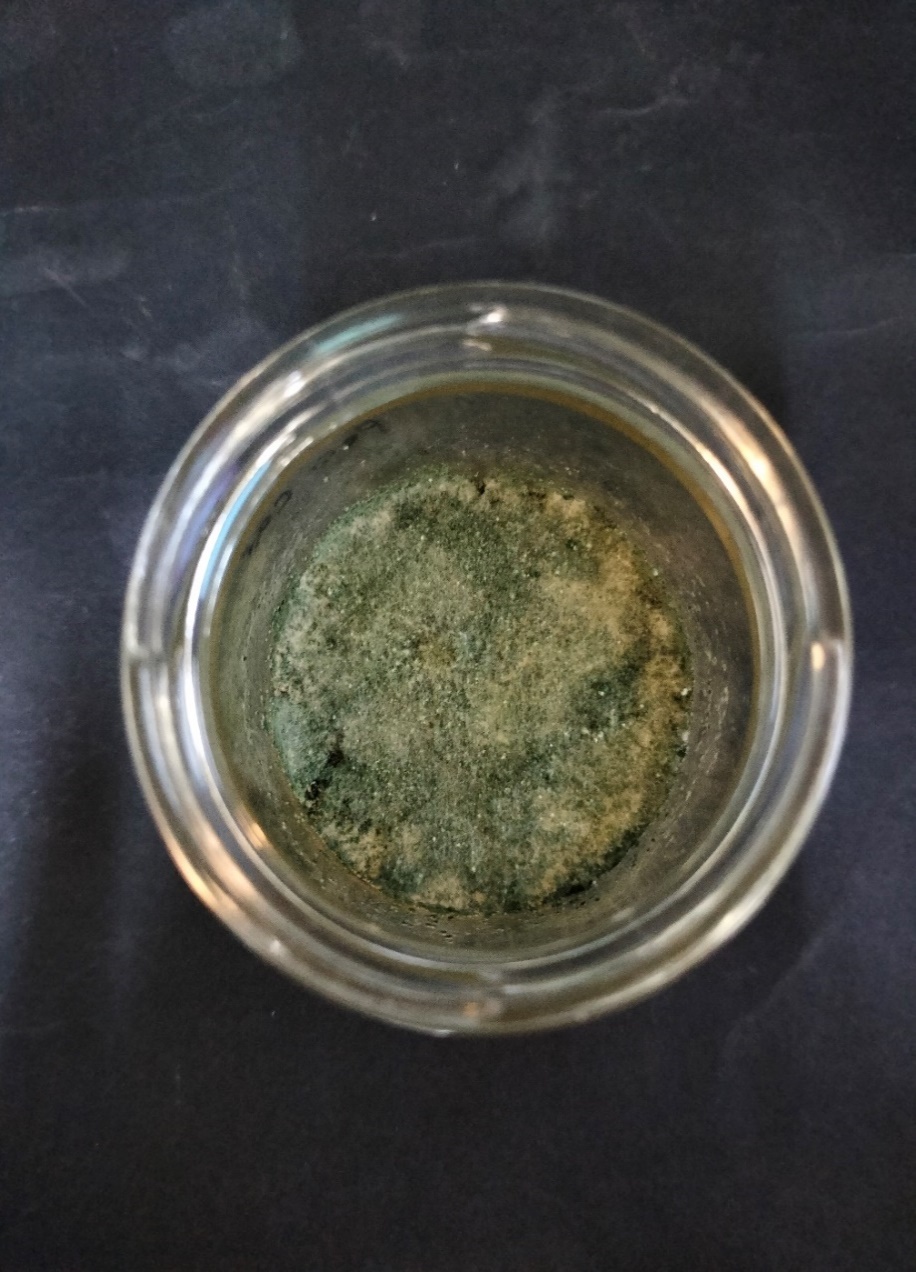


**Fig. S1.** *Trichoderma afroharzianum* isolate TRI07 growth morphology.

**
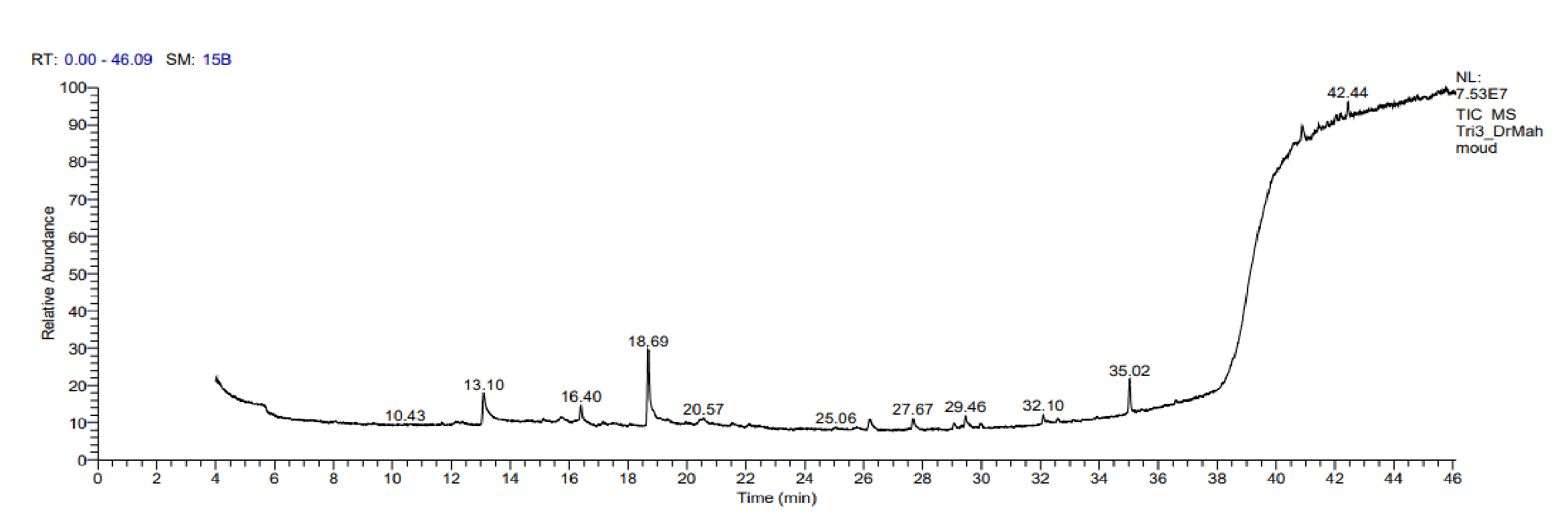
**

**Fig. S2.** GC-MS analysis for *T. afroharazianum* TRI07 ethyl acetate culture filtrate extract.

**Fig. S3** HPLC chromatographic charts resulted from tomato leaf extract analysis of different treatments. A1 = tomato plant control, A2 = plants inoculated by *A. alternata* (Alt3) only, A3 = plants inoculated with *T. afroharzinum* TRI07 48 h before inoculation with Alt3, A4 = plants inoculated with TRI07 after 48 h inoculation with Alt3**,** A5 = plants treated with fungicide 48 h before inoculation with Alt3, and A6 = plants treated with fungicide after 48 h of inoculation with Alt3**.**

**References**

1 Doley, K. & Jite, P. K. Interaction effects of Glomus fasciculatum and Trichoderma viride inoculations on groundnut plants inoculated with pathogen Macrophomina phaseolina. *IJAS* **4**, 281-288 (2014).

2 Rached-Kanouni, M. & Alatou, D. Change in activity of antioxidative enzymes in leaves of Acacia retinodes, Biota orientalis and Casuarina equisetifolia under heat stress condition. *European Scientific Journal* **9** (2013).

3 Beauchamp, C. & Fridovich, I. Superoxide dismutase: improved assays and an assay applicable to acrylamide gels. *Analytical biochemistry* **44**, 276-287 (1971).

4 VG, M. & Murugan, K. Antioxidant potentiality of partially purified protease inhibitor from the fruits of African nightshade (Solanum aculeatissimum Jacq.). *World J Pharm Pharm Sci* **2**, 5166-5181 (2013).

5 Srivastava, O. & Huystee, R. v. Evidence for close association of peroxidase, polyphenol oxidase, and IAA oxidase isozymes of peanut suspension culture medium. *Canadian Journal of Botany* **51**, 2207-2215 (1973).

6 Zhang, J. & Kirkham, M. Drought-stress-induced changes in activities of superoxide dismutase, catalase, and peroxidase in wheat species. *Plant and Cell Physiology* **35**, 785-791 (1994).

7 Shams Moattar, F., Sariri, R., Yaghmaee, P. & Giahi, M. Enzymatic and non-enzymatic antioxidants of Calamintha officinalis moench extracts. *Journal of Applied Biotechnology Reports* **3**, 489-494 (2016).

8 Velioglu, Y., Mazza, G., Gao, L. & Oomah, B. Antioxidant activity and total phenolics in selected fruits, vegetables, and grain products. *Journal of agricultural and food chemistry* **46**, 4113-4117 (1998).
